# A Novel Source of Cultured Podocytes

**DOI:** 10.1371/journal.pone.0081812

**Published:** 2013-12-12

**Authors:** Stefano Da Sacco, Kevin V. Lemley, Sargis Sedrakyan, Ilenia Zanusso, Astgik Petrosyan, Janos Peti-Peterdi, James Burford, Roger E. De Filippo, Laura Perin

**Affiliations:** 1 GOFARR Laboratory for Organ Regenerative Research and Cell Therapeutics, Saban Research Institute, Children’s Hospital Los Angeles; Department of Urology, University of Southern California, Los Angeles, California, United States of America; 2 Children’s Hospital Los Angeles, Division of Nephrology; Department of Pediatrics, University of Southern California, Los Angeles, California, United States of America; 3 Interdepartmental Research and Service Centre for Biology and Regenerative Medicine-Department of Pharmaceutical Sciences, University of Padova, Padova, Italy; 4 Department of Physiology and Biophysics, Zilkha Neurogenetic Institute, University of Southern California, Los Angeles, California, United States of America; Childrens Hospital Los Angeles, United States of America

## Abstract

Amniotic fluid is in continuity with multiple developing organ systems, including the kidney. Committed, but still stem-like cells from these organs may thus appear in amniotic fluid. We report having established for the first time a stem-like cell population derived from human amniotic fluid and possessing characteristics of podocyte precursors. Using a method of triple positive selection we obtained a population of cells (hAKPC-P) that can be propagated *in vitro* for many passages without immortalization or genetic manipulation. Under specific culture conditions, these cells can be differentiated to mature podocytes. In this work we compared these cells with conditionally immortalized podocytes, the current gold standard for *in vitro* studies. After *in vitro* differentiation, both cell lines have similar expression of the major podocyte proteins, such as nephrin and type IV collagen, that are characteristic of mature functional podocytes. In addition, differentiated hAKPC-P respond to angiotensin II and the podocyte toxin, puromycin aminonucleoside, in a way typical of podocytes. In contrast to immortalized cells, hAKPC-P have a more nearly normal cell cycle regulation and a pronounced developmental pattern of specific protein expression, suggesting their suitability for studies of podocyte development for the first time *in vitro*. These novel progenitor cells appear to have several distinct advantages for studies of podocyte cell biology and potentially for translational therapies.

## Introduction

The visceral epithelial cell (podocyte) is the pivotal cell maintaining normal structure and function of the kidney glomerulus [1]. Loss of podocytes is associated with progression of kidney disease in humans and experimental animals [2], [3] since there is at most a limited possibility to replace these post-mitotic cells [4]. When a sufficient number of nephrons are lost for any reason, progressive glomerular sclerosis ensues leading to total kidney failure.

The podocyte is a unique cell. Despite many characteristics typical of epithelial cells, its location, architecture and function are singular [4]. Attachment of podocyte foot processes to the glomerular basement membrane (GBM) makes direct isolation of podocytes difficult, so *in vitro* studies of these cells depend largely on cell culture systems.

The first cell culture work on podocytes was based on isolated primary podocytes [5] and suffered from several limitations, especially dedifferentiation of the cells over time. An important breakthrough was accomplished by Mundel *et al.* [6], and subsequently by Saleem *et al.* [7], when mouse and human conditionally-immortalized podocyte lines were created by expression of a temperature-sensitive mutant of the SV40 large-T antigen, allowing significant advances in our understanding of podocyte cell biology. For example, *in vitro* studies using immortalized podocytes [8], [9] have suggested podocyte-specific mechanisms for some drug-based therapies for the nephrotic syndrome previously thought to act only via the immune system. Despite the widespread use of conditionally immortalized podocytes in research, some limitations of these cells (like sensitivity of the cell phenotype to culture conditions [10] or dramatic differences in phenotype among different podocyte lines [11]), suggest the need to develop novel *in vitro* podocyte culture systems.

Amniotic fluid (AF) is in continuity with multiple developing organ systems, including the kidney. Committed, but still stem-like cells from these organs may thus appear in AF.

In this work, we isolated and characterized a novel cell population derived from human amniotic fluid cells, possessing characteristics of podocyte precursors (Amniotic-fluid Kidney Progenitor Cells-Podocytes, hAKPC-P).

We compared differentiated hAKPC-P with human immortalized podocytes (hIPod). After *in vitro* differentiation, these cells have characteristics similar to immortalized podocyte cell lines: expression of the major podocyte proteins including mature (α3α4α5) type IV collagen, a typical response to podocyte toxins, and physiological and morphological properties that resemble *in vitro* podocytes. In contrast to existing immortalized cell lines, this cell population can be cultured from any mammalian model system and propagated for many passages without immortalization, and has a more nearly normal cell cycle regulation as well as a clear developmental pattern of specific protein expression, possibly allowing studies of podocyte development *in vitro*. Thus, culture systems derived from these unique progenitor cells may represent an attractive alternative to current immortalized cell lines.

## Materials and Methods

### Ethics Statement

Samples of human amniotic fluid from male fetuses (15–20 weeks of gestation) were provided to our laboratory by Labcorp (Monrovia, CA, USA) after karyotyping analysis. Immortalized podocytes were kindly donated by Dr. J. Reiser and were cultured as reported by Saleem *et al.* [7] Approval of the study, as well as written or verbal consent was not required since samples were not identified and information obtained about the samples was limited to karyotype and fetal health status (45 CFR 46.102). Based on these facts, and after a detailed review, the requirement for an approval was waived by the Children’s Hospital Los Angeles Institutional Review Committee/Committee of Clinical Investigation (IRB/CCI).

Whole kidneys were isolated from adult C57/BL6 mice in adherence to the National Institutes of Health Guide for the Care and Use of Laboratory Animals and performed in accordance with protocols approved by the Institutional Animal Care and Use Committee at Children’s Hospital Los Angeles.

### Isolation and Cell Culture of hAKPC-P, hIPod and Podocyte Differentiation and Controls

Four human amniotic fluid samples with normal male karyotypes and normal fetal ultrasounds were collected from amniocenteses between 15–20 weeks of gestation and kindly donated by Dr R. Habibian (Labcorp, Monrovia, CA). Cells were expanded in Chang’s medium [12], [13] (alpha-MEM, 20% Chang-B, 2% Chang-C) (IrvineScientific), L-Glutamine, 20% ES-FBS, and 1% antibiotic (Gibco/Invitrogen). Total amniotic fluid population cells were labeled with anti-CD24 (1 µg/ml, Abcam), anti-OB-Cadherin (1 µg/ml, Abcam) and anti-podocalyxin (1 µg/ml, Abcam) antibodies marked with Zenon kit fluorochromes (Alexa 488,647 and R-PE, Invitrogen). Cells were sorted using a FacsAria flow cytometer (BD Biosciences). Sorted cells were also expanded in Chang medium. hIPod were cultured as described by Saleem *et al*. [7]. Differentiation of hAKPC-P was performed by culturing them on collagen I coated plates in VRADD media [RPMI-1640 supplemented with 10% FBS, 1% antibiotic, 1,25(OH)2D3 (100 nM), ATRA (1 µM), dexamethasone (100 nM), 1× insulin-transferrin-selenite (ITS)] for up to 30 days. Re-differentiation of hIPod was performed by thermoshifting to 37° for up to 15 days.

Human bone marrow mesenchymal cells (BM-MSCs), obtained from LifeLine Cell Technology were expanded using standard protocols. Briefly, the cells were cultured in DMEM/F12 (Life Technologies), 10% FBS (Life Technologies), 1% Penicillin/Streptozotocin (Life Technologies) in tissue culture dishes and passaged every 3 days. Human Lung fibroblasts were purchased from LifeLine Cell Technology and expanded with Fibrolife Media (LifeLine Cell Technology) in tissue culture dishes for up to 5 passages. When cultured in VRADD media, fibroblasts died within a day. Mouse kidney cortex cells were used in the FACS analysis. Briefly, a single cell suspension was obtained from mouse kidney cortex digested with dispase II (Sigma-Aldrich) for 2 hours [13]. Then the suspension was washed thoroughly with PBS and fixed with 4% paraformaldehyde (PFA, Polysciences), followed by permeabilization with 0.1% saponin (Sigma-Aldrich) for 10 minutes.

### Population Doubling Times

Measurement of population doubling times for undifferentiated hAKPC-P and hIPod was performed by seeding 500,000 cells under the culture conditions described above and counting the number of cells after 24 hours, 48 hours and 72 hours. To calculate the doubling time, the following equation was used (as described on “Basic cell culture: a practical approach” edited by J.M. Davis, 2001, Oxford University Press).
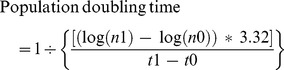



*Cell size, percentage of multi-nucleated cells and assessment of cell-cell contacts.* To calculate cell size, tiff images were obtained with an AMG Evos Microscope and analyzed with Adobe Photoshop CS5. A given cell’s size was defined along its border by the use of the “magnetic lasso tool”. The pixel value of the total selected area was calculated using the “Histogram” panel and then recorded in an Excel spreadsheet. To determine changes in cell size after differentiation, at least 200 measurements of apparent cell area were performed for each group (undifferentiated and differentiated) for both hIPod and hAKPC-P. Cell size (defined as mean number of pixels per measurement) was then normalized to the mean value for the undifferentiated cells of that line. Data are expressed as mean ± SEM. The percentage of multinucleated podocytes was assessed by counting multinucleated cells present in images (20X) randomly acquired across the tissue culture dish. To calculate the percentage of foot process contacts we randomly chose three SEM pictures (magnification 1800x–2000x, for both hAKPC-P and hIPod). We counted the percentage of foot processes that formed contacts with neighboring foot processes compared to the total number of foot processes extending from the body cell.

*Western blotting, transmission electron microscopy* (*TEM*), scanning electron microscopy (*SEM), immunostaining, H&E staining* were performed as previously described [12], [13] using standard protocols. Mouse kidney lysate, was obtained as previously described [13] and used as control for western blotting experiments. In particular for western blotting of the alpha chains of the type IV collagen, because of the technical requirements of the preparation, the beta-actin extraction had to be done in separate, equivalent samples and thus is a representative sample for the loading control. Antibody concentrations and dilutions are described in Supplementary Table S1 in Supplementary File S1. For TEM, samples were processed by the Pathology laboratory at Children’s Hospital Los Angeles. For SEM, samples were processed at the University of Southern California Keck School of Medicine microscopy core. For quantification of foot processes, 50 cells (both for hAKPC-P and hIPod) were observed in SEM.

### Microarray Analysis

RNA was extracted from 3 samples each of undifferentiated hAKPC-P, differentiated hAKPC-P, de-differentiated hIPod and re-differentiated hIPod, using the Qiagen RNAeasy kit, and quantitated using a NanoDrop 1000 Spectrophotometer (ThermoScientific). RNA integrity number (RIN) was obtained using the RNA 6000 Nano Chip on the Agilent Bioanalyzer (Agilent) and only samples with RIN values higher than 7 were analyzed. RNA was labeled using Ambion WT Expression Kit (Life Technologies) and cDNA produced. Labeled samples were hybridized to Affymetrix Human Gene 1.0 ST arrays. Array data were imported into Partek genomic Suite 6.6 (Partek Incorporated) and quantile normalized using robust multichip averaging (RMA). Following normalization, log2 expression signals from probe sets were summarized in a data table. Ingenuity Pathways Analysis (IPA) (Ingenuity Systems, www.ingenuity.com) of microarray data was used to identify significant differences (P<0.05) in expression of genes between de-differentiated and re-differentiated hIPod and undifferentiated and differentiated hAKPC-P. Fold variation between groups were calculated using median values. t-tests were performed to assess significant values. Symbols with the same shape (oval, circle, diamond, etc.) share a similar function. The data discussed in this publication have been deposited in NCBI’s Gene Expression Omnibus and are accessible through GEO Series accession number GSE49439.

### Decellularization of Mouse Glomerular Tufts, Cell Seeding and Cell Counting

Whole kidneys were isolated from adult C57/BL6 mice in accordance with protocols approved by the Institutional Animal Care and Use Committee at Children’s Hospital Los Angeles. Kidneys were removed from their capsule and rinsed twice in PBS. Renal acellular matrices were prepared by the Meezan method [14] performing four decellularization cycles, while the decellularized glomerular tufts were isolated following the method of Takemoto *et al.* [15]. The lack of cells or cell debris was confirmed histologically (H&E and DAPI staining) and by SEM. Before seeding of the tufts, hAKPC-P and hIPod were differentiated for 7 days in their respective differentiation media. Subsequently, after trypsinization, 300,000 hAKPC-P or hIPod were seeded onto the tufts and incubated at 37°C for additional 21 days to reach full differentiation. hFibroblasts were seeded in the same number and cultured under normal conditions using Fibrolife medium (when fibroblasts are cultured in podocyte differentiation media, they die within a day). Samples were prepared for SEM analysis or fixed in 10% phosphate buffered formalin, embedded in paraffin, sectioned at 5 µm and stained with H&E or DAPI for histological analysis. To assess the number of cells per glomerular tuft, 8 paraffin sections (5 µm, H&E stained) from different seeding experiments (hIPod and hAKPC-P) were randomly picked for analysis. To calculate tuft cross-sectional area, tiff images were obtained with an AMG Evos Microscope and analyzed with Adobe Photoshop CS5. A given tuft area size was defined along its border by the use of the “magnetic lasso tool”. The pixel value of the total selected area was calculated using the “Histogram” panel and then recorded in an Excel spreadsheet. Number of nuclei per tuft cross-sectional area was determined for a single section through each tuft, giving an estimate of the nuclear density of cells in the tufts. Data are expressed as value + SEM.

### Calcium Intake in Response to Flufenamic Acid (FFA) and Angiotensin II (Ang II)

hIPod and hAKPC-P cells were grown on circular glass coverslips (25 mm; VWR). During Ang II or FFA experiments, cells were superfused at 1 ml/min with a modified Krebs-Ringer-HCO_3_ buffer containing (in mM) 115 NaCl, 5 KCl, 25 NaHCO_3_, 0.96 NaH_2_PO_4_, 0.24 Na_2_HPO_4_, 1.2 MgSO_4_, 2 CaCl_2_, 5.5 D-glucose, and 0.1 L-arginine. All solutions were adjusted to pH 7.4 by bubbling with a 5% CO_2_ and 95% O_2_ gas mix. Time-lapse ratiometric calcium imaging was performed with a Leica TCS SP2 AOBS MP confocal microscope system (Leica Microsystems) using the dyes Fluo-4 (excitation at 488 nm, emission at 520±20 nm) and Fura red (excitation at 488 nm, emission at >600 nm). A transmitted light detector and differential interference contrast (DIC) imaging was used to measure cell fluorescence. All experiments were performed using the same instrument settings (laser power, offset, gains of both detector channels). Data acquisition and analysis was done using the Leica LCS imaging software LCS 2.61.1537. Cells were loaded with the calcium dyes Fluo-4 AM and Fura red AM (10 µM each; Invitrogen) dissolved in DMSO, in modified Krebs-Ringer-HCO3 at room temperature for approximately 20 minutes. After incubation, coverslips were transferred to the chamber of the Leica confocal microscope system for imaging with a 40× oil-immersion objective. Fluo-4/Fura red ratios used to indicate [Ca^2+^]_i_ were calculated using the LCS software. The following pharmacological agents were used in the experiments: FFA (200 µM, Sigma), and Ang II (1 nM, Phoenix Pharmaceuticals). Both pharmacologic agents were added to the chamber superfusion (bathing) solution.

### Contractility after Ang II Stimulation and Cell Size

Differentiated hAKPC-P and hIPod were starved of serum for 2 hours before stimulation with Ang II 10^−5^ M (Sigma-Aldrich). To calculate cell size, tiff images were obtained, and analyzed with Adobe Photoshop CS5 as previously described. Measurements of cell size area after Ang II stimulation were performed for each group as previously described. Data are expressed as mean + SEM.

### Cell Cycle Analysis

Cell cycle analysis was performed for both dedifferentiated and re-differentiated hIPod and undifferentiated and differentiated hAKPC-P. For de-differentiation experiments hIPod were transferred to permissive conditions (33°C). Undifferentiated hAKPC-P cells were kept at 37°C and cultured with RPMI 1640, 10% FBS, 1% Penicillin/Streptomycin and ITS 1X.

Cells were trypsinized, washed and fixed in 70% ethanol at 4°C for one hour. Cells were then resuspended in PBS (Sigma) containing propidium iodide (PI, 20 µg/ml, Sigma). Cell cycle distribution was analyzed by flow cytometry (FacsCalibur, BD Biosciences). Data from 40,000 cells per sample were collected and analyzed using ModFit (Verity Software House).

### Analysis of Percentage of Nephrogenic Progenitor and Podocyte Protein Expressing Cells by FACS

Undifferentiated hAKPC-P, de-differentiated hIPod, lung fibroblasts and mouse cortex renal cells were stained for CD24 (Abcam), OB-Cadherin (Abcam), Podocalyxin (Invitrogen), WT1 (R&D Systems) and Nephrin (Thermo Scientific) at a concentration of 1 µg/ml unless otherwise specified in the datasheet. Additionally, undifferentiated hAKPC-P were stained for Six2 (Santa Cruz) and Cited1 (Novus Biological) at a concentration of 1 µg/ml unless otherwise specified in the datasheet. Data acquisition and analysis of all samples by flow cytometry was performed with a BD FACSCalibur flow cytometry system (BD Biosciences). Data analysis was performed with FlowJO.

### Statistical Analysis

All graphical data are displayed as the mean + SEM. Mann-Whitney U test was applied when normality of distributions did not obtain. ANOVA was used to compare independent sets of normally distributed data. A P value less than 0.05 was considered statistically significant.

## Results

### Isolation of hAKPC-P

**Figure 1:**
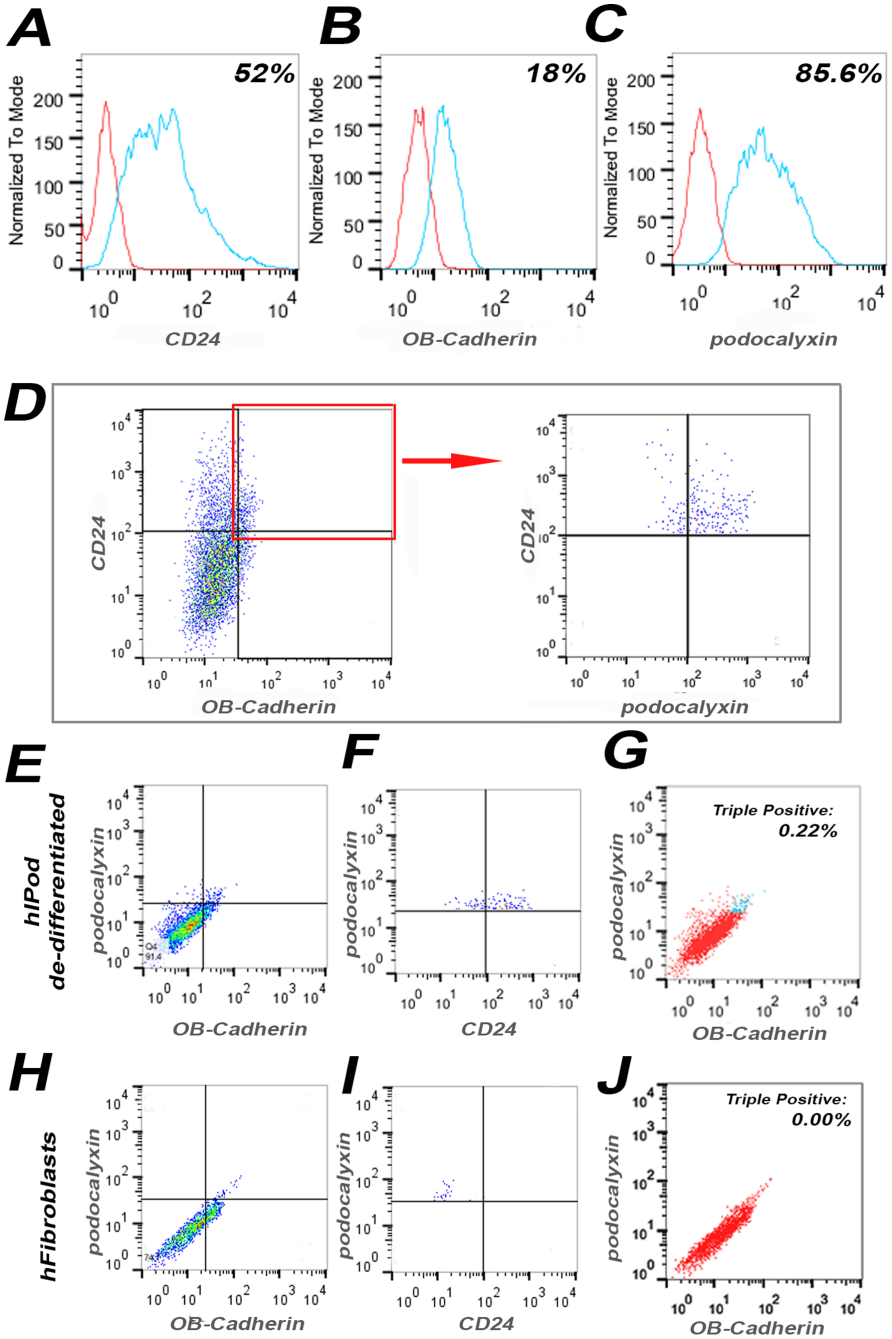
Isolation of hAKPC-P. FACS analysis of human amniotic fluid cells for CD24 (**A**), OB-Cadherin (**B**) and podocalyxin (**C**). hAKPC-P cells were isolated by FACS sorting amniotic fluid samples from weeks 15 to 20. About 4–10% of the total amniotic fluid cell population (hAKPC) co-expressed CD24 and OB-Cadherin while triple-positive cells (hAKPC-P) accounted for about 0.5–1.6% of the total population (**D**). **E–J** FACS analysis showing percentage of cells triple positive for CD24, OB-Cadherin and podocalyxin for hIPod de-differentiated (**E–G**, 0.22% triple positive cells) and hFibroblasts (**H–J**, negative control, no triple positive cells). (Red line = unstained sample; Blue line = stained sample).

We have already demonstrated that human amniotic fluid harbors cells expressing a variety of lineage-specific markers, including renal markers [12]. Among other markers, subpopulations of amniotic fluid cells express CD24, OB-Cadherin and podocalyxin (Fig. 1A–C). Triple selection for these markers identified a specific precursor cell population (Fig. 1D), representing 0.5–1.6% of all amniotic cells (hAKPC-P). In order to characterize the specificity of our selection method, we compared hAKPC-P to human immortalized podocytes [7] (hIPod), human lung fibroblasts (hFibroblasts, negative control), mouse kidney cortex cells (mKC, additional control) and human bone marrow mesenchymal stem cells (hBM-MSCs, as a different stem cell source) (Fig. 1, Fig. S1–S2). hIPod had 0.22% triple positive cells before re-differentiation (Fig. 1E–G) and mKC had 0.02% triple positive cells (Fig. S1A–C). Both hBM-MSCs (Fig. S1D–F) and hFibroblasts (Fig. 1H–J) contained cells positive for CD24, OB-Cadherin and podocalyxin individually, but neither population contained cells expressing all three markers. Therefore, we decided to further study only hFibroblasts as a negative control. Moreover only 0.01% of the hAKPC-P after differentiation (Fig. S1G–I) and 1.77% of the hIPod after re-differentiation (Fig. S1J–L) expressed all three markers. The expression of each marker singularly (CD24, OB-Cadherin and podocalyxin) for the different populations (hAKPC-P before and after differentiation, hIPod before and after re-differentiation, hBM-MSCs, hFibroblasts and mKC) by FACS analysis is reported in Fig. S2A–R. Based on this expression pattern, we hypothesize that hAKPC-P represents podocyte precursors cells.

### hAKPC-P Acquire Specific Morphological and Phenotypical Traits of Mature Podocytes after Differentiation

In this work we compared *in vitro* hIPod with hAKPC-P. We differentiated both cell types into mature podocytes: hIPod were differentiated at the non-permissive temperature (37°C) as described by Saleem *et al.* [7], while hAKPC-P were differentiated by culture in a defined medium [16].

**Figure 2:**
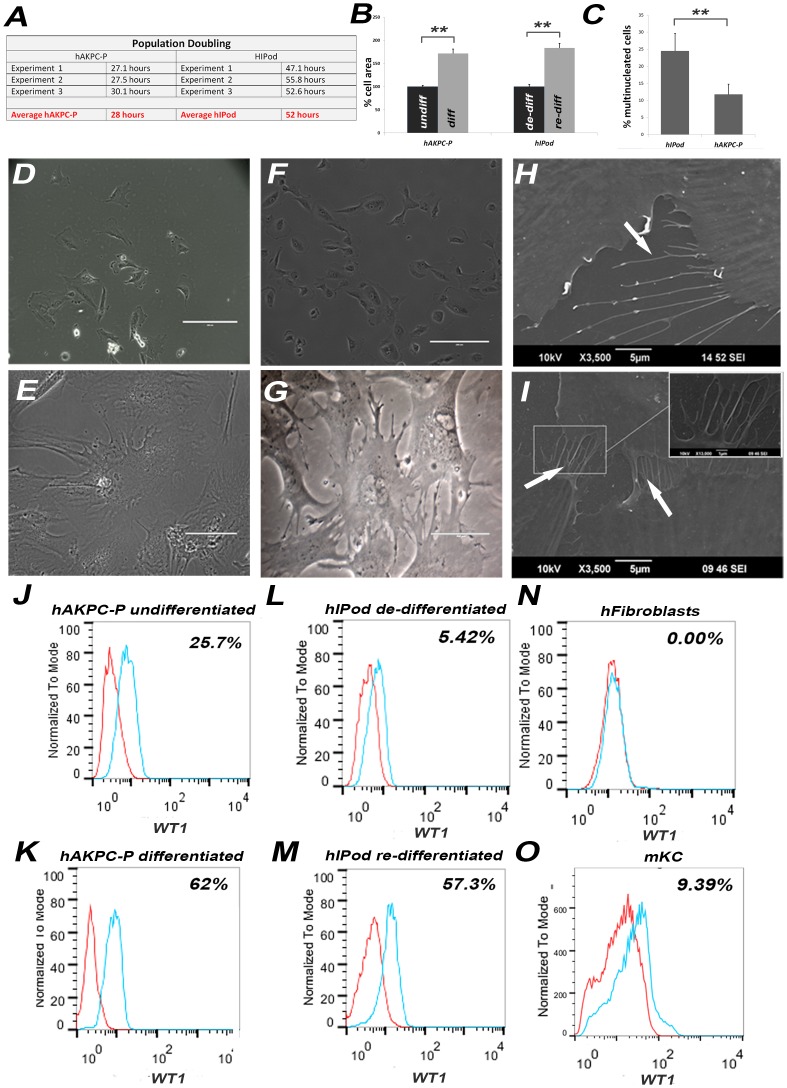
Morphological and phenotypical analysis of hAKPC-P and hIPod before and upon differentiation. A. hAKPC-P before differentiation had an average population doubling time of 28 hours while hIPod had a doubling time of 52 hours. **B**. Upon differentiation, there was an increase in apparent cell size of both hAKPC-P and hIPod (expressed as percentage of baseline cell area). Values are presented as mean + SEM (** = P<0.01). **C**. The percentage of multinucleate cells in hIPod was greater than in hAKPC-P upon differentiation. Values are presented as mean ± SEM (P<0.05, Mann-Whitney U). **D–E** Representative pictures demonstrating the morphology of hIPod at permissive temperature (**D**) and after re-differentiation at non-permissive temperature (**E**). **F–G**. Representative pictures of hAKPC-P in culture before differentiation (**F**) and under differentiating conditions showing numerous primary processes (**G**). Scale bar = 200 µm. **H–I**. Representative scanning electron micrographs (SEM) of hIPod (**H**) and hAKPC-P (**I**) after differentiation. Processes extending from cell body (arrows; scale bar = 5 µm) and cell-contacts are evident (magnified insert, scale bar = 1 µm). **J–O** FACS analysis showing the percentage of cells positive for WT1 among hAKPC-P and hIPod before (**J, L**) and after (**K, M**) differentiation and compared to hFibroblasts (**N**) and cells from mouse kidney cortex, mKC (**O**). (Red line = unstained sample; Blue line = stained sample).

Before differentiation, hAKPC-P had a population doubling time of 28 hours, while that of hIPod was 52 hours (Fig. 2A), indicating that hAKPC-P can be expanded in less time. In the undifferentiated state, hAKPC-P grow easily in culture and are capable of extensive self-renewal, since they were passaged more than 50 times while maintaining viability and the capacity for differentiation.

Shankland and colleagues have provided criteria for classifying cultured cells as podocytes [10]. We compared hAKPC-P and hIPod in light of these criteria. Upon differentiation, both populations increased their apparent cell size (Fig. 2B), produced multinucleate cells (Fig. 2C), and changed their morphology (Fig. 2D–G), developing primary processes in all cells (Fig. 2H–I) – all characteristics of cultured podocytes [4], [10]. In particular, cultured cells formed secondary processes extending from the cell body. These had apparent dimensions of foot processes (≈400 nm) [17] and formed contacts with neighboring cells (Fig. 2H–I). Cell-cell interaction occurred more commonly in hAKPC-P (61.8%±4.9 SEM) than in hIPod (45.2%±9 SEM).

We also evaluated the differentiation capacity of both hAKPC-P and hIPod, using the expression of WT1, which is expressed by mature podocytes *in vivo* [10] but is also relevant to podocyte specification during development [18]. FACS analysis, revealed increased expression of WT1 after differentiation in both hAKPC-P and hIPod cell populations (62%, 57.3%), while hFibroblasts showed no expression of WT1, and mKC had less than 10%, respectively (Fig. 2J–O).

### Cell-cell Interaction in Differentiated hAKPC-P and Expression of Podocyte-specific Proteins

**Figure 3:**
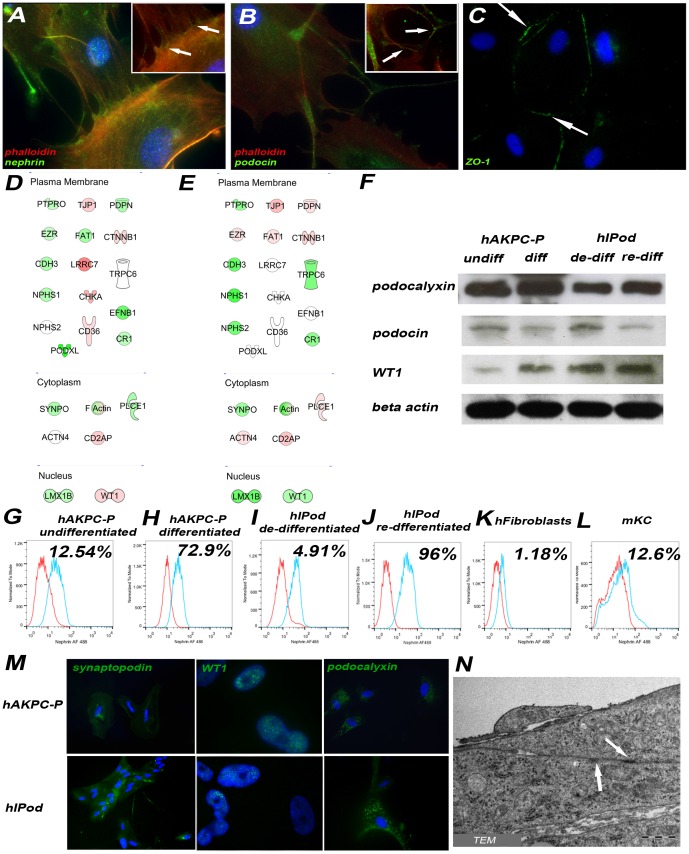
Analysis of expression of specific podocyte markers, slit diaphragm protein expression and adherens-type junctions in differentiated hAKPC-P and re-differentiated hIPod. *A*. Differentiated hAKPC-P formed cell-cell contacts containing nephrin (green). Phalloidin staining (red) identifies the actin cytoskeleton; nuclei are stained blue (DAPI) in this and subsequent micrographs (40X; magnified insert 100X; arrows indicate cell-cell contacts) **B.** hAKPC-P also expressed podocin (green, 40X; magnified insert 100X; arrows indicate protein expression along processes). **C.** hAKPC-P expressed ZO-1 (green) along cell-cell contacts (arrows, 40X). **D–E.** Ingenuity Pathways Analysis (IPA) of microarray data was used to identify significant differences in expression of genes fundamental to podocyte maturation between undifferentiated hAKPC-P and de-differentiated hIPod (**D,** Table S2 in File S1) and between differentiated hAKPC-P and re-differentiated hIPod (**E,** Table S2 in File S1). In particular, differentiated hAKPC-P express higher RNA levels of LMX1B, WT1, NPHS1, NPHS2 and SYNPO compared to re-differentiated hIPod. Red symbols signify a higher mRNA content of hIPod, while green symbols signify a higher mRNA content in the hAKPC-P. Only significant differences (P<0.05) in gene expression are reported. Symbols with the same shape (oval, circle, diamond, etc.) share a similar function. **F.** Western blotting analysis for podocalyxin (160 kDa), podocin (42 kDa) and WT1 (51 kDa) in both hAKPC-P and hIPod after differentiation (housekeeping gene: beta-actin). **G–L.** FACS analysis for nephrin in undifferentiated (**G**) and differentiated (**H**) hAKPC-P, de-differentiated hIPod (**I**) and re-differentiated hIPod (**J**) confirming an increase in the percentage of cells expressing nephrin in hAKPC-P and hIPod after differentiation. Only 1.18% of the hFibroblasts were positive for nephrin (**K**) while about 12.6% of the cells from the mouse kidney cortex, mKC, showed positivity (**L**). (Red line = unstained sample; Blue line = stained sample). **M.** Representative immunofluorescence for synaptopodin (green, 40X), WT1 (green, 100X) and podocalyxin (green, 40X) in hAKPC-P and hIPod after differentiation. **N.** Representative transmission electron micrograph (TEM) showing apparent adherens-type junction structures between differentiated hAKPC-P (arrows, scale bar = 1 µm).

The slit diaphragm (SD) associated proteins (ZO-1, podocin and nephrin [19]) along with other proteins like synaptopodin, GLEPP1 and TRPC6 are expressed in mature podocytes *in vivo* and *in vitro*. We observed that both hAKPC-P and hIPod express these proteins and their associated mRNAs after differentiation (Fig. 3A–M; Fig. S3, Fig. S4A). Importantly, the microarray data show an increase of podocyte-specific mRNAs associated with the differentiation process in hAKPC-P; in particular for *GLEPP1 (PTPRO), NPHS1, NPHS2, TRPC6, LMX1B, SYNPO, WT1,* and the collagen IV α-chains (*COL4A3*, *COL4A4*, *COL4A5*). On the other hand, hIPod do not show strongly increased expression of these genes after being re-differentiated (Fig. 3D–E).

We observed that hAKPC-P form SD-like structures, characterized by strong expression of nephrin, podocin and ZO-1 at cell-cell contacts (Fig. 3A–C) after differentiation. In addition, staining for nephrin was evident at cell-cell contacts in hAKPC-P and in hIPod (Fig. 3A arrows and Fig. S3i arrows). Typical adherens junction-like structures [20] were visible at cell membrane contacts between differentiated hAKPC-P by electron microscopy (Fig. 3N). The staining results for nephrin, podocin, ZO-1, synaptopodin, WT1 and podocalyxin for undifferentiated hAKPC-P, de-differentiated, re-differentiated hIPod and hFibroblasts are shown in Fig. S3A–Y.

These data show that hAKPC-P had a clear developmental pattern of expression of podocyte genes and proteins, while hIPod lack of this developmental phenotype, suggesting that the “re-differentiation” process in hIPod under non-permissive conditions does not completely recapitulate the differentiation process of normal podocyte development.

### Extracellular Matrix Production and Cell – Matrix Interactions in Differentiated hAKPC-P

In addition to interactions with neighboring podocytes, podocytes interact with their underlying GBM, including producing mature type IV collagen (α3α4α5) and laminin 521, major constituents of the GBM [4].

**Figure 4:**
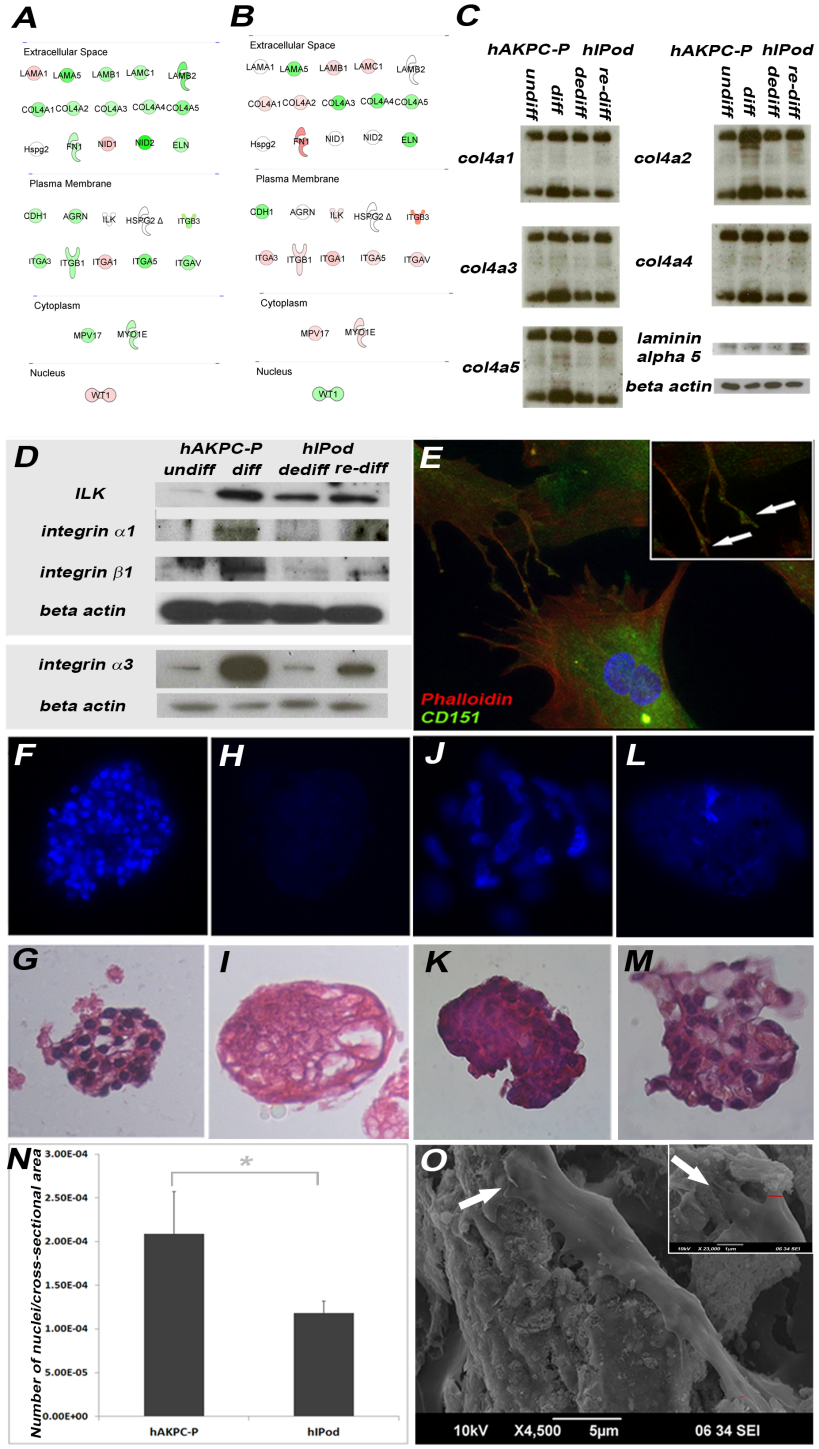
Glomerular basement membrane protein expression, integrin-ILK expression and attachment to decellularized glomerular tufts by hAKPC-P and hIPod. A–B Ingenuity Pathways Analysis (IPA) of microarray data was used to identify significant differences in expression of genes fundamental to GBM formation between undifferentiated hAKPC-P and de-differentiated hIPod (**A,** Table S3 in File S1) and between differentiated hAKPC-P and re-differentiated hIPod (**B,** Supplementary Table S3 in File S1). Red symbols signify a higher mRNA content of hIPod, while green symbols signify a higher mRNA content in the hAKPC-P. Only significant differences (P<0.05) in gene expression are reported. Symbols with the same shape (oval, circle, diamond, etc.) share a similar function. **C.** The western blot analysis confirmed protein expression of collagen IV α1, α2, α3, α4, and α5 chains in both cell lines before and after differentiation. Under reducing conditions, collagen IV α-chains exist in both monomeric (25 kDa) and dimeric (50 kDa) forms. Laminin α5 (37 kDa) is expressed in both cell lines before and after differentiation (housekeeping gene: beta-actin). **D.** Western blot analysis showed that ILK (51 kDa), integrin α3 (131 kDa) and integrin β1 (188 kDa) in both lines before and after differentiation (housekeeping gene: beta-actin). **E.** Representative picture of hAKPC-P after differentiation expressing CD151 (green), which is also present in cell processes (100X). **F–M.** Representative images of isolated murine glomerular tufts prior to and after decellularization and seeding with hAKPC-P or hIPod. Before decellularization, glomeruli had numerous nuclei as shown by DAPI (**F**) and H&E (**G**) staining. After decellularization of the murine glomerulus, the absence of nuclei was tested by DAPI (**H**) and H&E (**I**) (40X). Seeded hAKPC could be seen by DAPI (**J**) or H&E (**K**); similarly for hIPod (**L–M**). The difference in nuclear size is obvious between mouse (**F, G**) and human (**J, M**) cells (all images at 40X). N. Graph representing the number of nuclei (hAKPC-P and hIPod) per tuft cross-sectional area (n = 8 each) 5 days after seeding, showing that glomerular tufts seeded with hAKPC-P have a higher density of adherent cells than hIPod (arbitrary area units). Values are presented as mean + SEM (P<0.05, Mann-Whitney U). **O.** Representative scanning electron micrographs (SEM) of a decellularized glomerular tuft seeded with hAKPC-P. Numerous processes extending from the cell body (arrows, 4500X) were evident having widths of 300–400 nm (higher power insert, red lines).

We detected proteins for all type IV collagen chains before and after differentiation in both cell lines (Fig. 4C), although a larger increase in protein expression was apparent in hAKPC-P. Microarray data in Fig. 4A–B in hAKPC-P also showed a clear increase in gene expression of *COL4A3*, *COL4A4* and *COL4A5*, and both lines produce message for laminin α5 after differentiation. hFibroblasts were negative for collagen IV alpha chains while mouse kidney lysate showed expression of the type IV collagen proteins (Fig. S4B).

In differentiated hAKPC-P, in contrast to hIPod, we detected strong expression of both the α3 and β1 integrin chains (Fig. 4D). Only hAKPC-P showed a significant increase in *ILK* protein with differentiation, suggesting another developmental process in this cell line.

Microarray data (Fig. 4B) show that, when compared with differentiated hAKPC-P, re-differentiated hIPod expressed other types of integrins (β3, α5, αV) that are necessary for adhesion to fibronectin-containing substrates [21]. Expression of the αVβ3 integrin dimer may represent a stress response of immortalized podocytes to stimuli such as mechanical stretch [21]. This suggests that hIPod produce integrins and extracellular matrix components different from the native GBM components of hAKPC-P and *in vivo* podocytes. In addition, differentiated hAKPC-P and re-differentiated hIPod expressed CD151 (Fig. 4E, Fig. S3L), while it was not found in undifferentiated hAPKPC-P, de-differentiated hIPod and hFibroblasts (Fig. S3D,H,P). Expression of CD151 is also characteristic of podocyte specification, since its interaction with integrin α3β1 is involved in podocyte binding to laminin 521 in the GBM [22].

To test whether cultured podocytes are able to attach to extracellular matrix of the glomerular tuft, we isolated (Fig. 4F–G) and decellularized (Fig. 4H–I) glomeruli from adult mouse kidneys and seeded them with either hAKPC-P or hIPod, previously cultured for 7 days under differentiation conditions. The decellularized glomeruli were repopulated better by hAKPC-P (Fig. 4J–K) than by hIPod (Fig. 4L–M, N). We also observed that differentiated hAKPC-P growing on the decellularized glomerular tufts made contact with the matrix and exhibited numerous processes extending from the cell body and having widths suggesting podocyte foot processes [17] (Fig. 4O). Such processes were not observed with hIPod, suggesting that hAKPC-P have a greater capability to attach onto a natural scaffold and seem to approximate some aspects of an *in vivo* podocyte phenotype under these circumstances. When hFibroblasts were seeded onto decellularized glomerular tufts, they didn’t show the ability to repopulate the decellularized glomeruli, and in fact seemed to digest the tufts (data not shown).

### Differentiated hAKPC-P show Specific Functional Traits of Mature Podocytes

**Figure 5:**
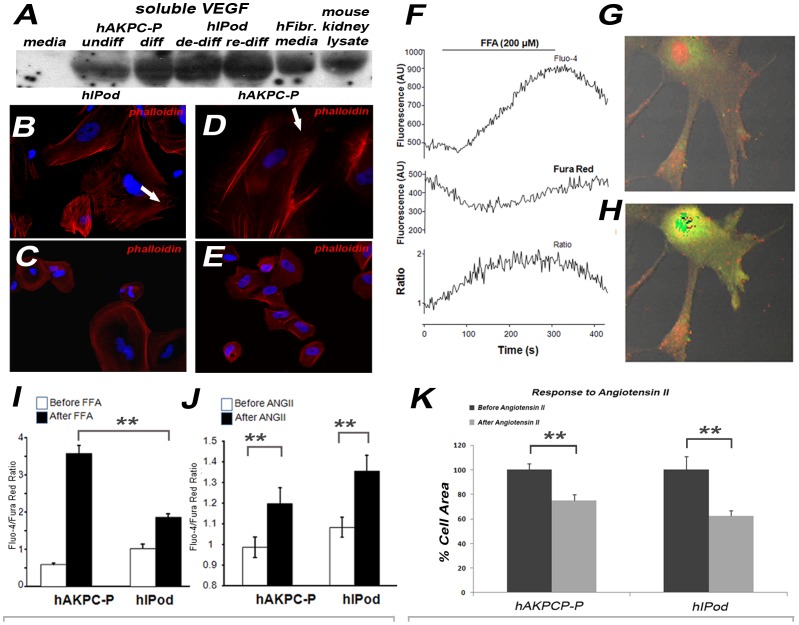
Functional analysis of differentiated hAKPC-P and hIPod: VEGF production, PAN toxicity and Ca^2+^ uptake. A. Representative Western blotting of soluble vascular endothelial growth factor (VEGF) in culture media from hAKPC-P and hIPod before and after differentiation compared with hFibroblasts and their corresponding media and mouse kidney lysate. **B–E.** Representative images of the podocyte actin cytoskeleton before (**B,D**) and after (**C,E**) exposure to PAN. Note loss of stress fibers and formation of cortical actin after PAN. 40X. **F–J.** Ratiometric fluorescence imaging of changes in cytosolic calcium ([Ca^2+^]_i_) in cultured hAKPC-P and hIPod in response to FFA (200 µM) or Ang II (1 nM) using the calcium-sensitive fluorochromes, Fluo-4 and Fura Red **F**: Administration of FFA, a TRPC6 agonist, to the culture superfusate caused a robust, transient elevation in the Fluo-4/Fura Red fluorescence intensity ratio, indicating an increase in [Ca^2+^]_i_. **G–H**: Representative images of hAKPC-P before (**G**) and after (**H**) FFA administration. DIC overlay of Fluo-4 (green) and Fura Red (red) is shown. **I**: hAKPC-P cells showed significantly greater FFA-induced increases in [Ca^2+^]_i_ (estimated by changes in Fluo-4/Fura Red fluorescent ratio before and after FFA administration ) compared to hIPod (n = 8 each). **J**: both hAKPC-P and hIPod showed significant and equivalent elevations in [Ca^2+^]_i_ in response to 1 nM ANGII (n = 9 each); the y-axis is Fluo-4/Fura ratio. Values are presented as mean ± SEM (** = P<0.01). **K.** Following Ang II stimulation, both cell lines contracted, with decreases in apparent cytoplasmic area. Values are presented as mean + SEM (** = P<0.01).

As proposed by Shankland *et al.* [10], in order to be classified as a podocyte in culture, in addition to specific protein expression, a cell should have some important functional characteristics, such as secretion of vascular endothelial growth factor (VEGF), a typical response after exposure to toxins such as puromycin aminonucleoside (PAN), and formation of processes in response to all-trans retinoic acid (ATRA). In differentiated hAKPC-P, we observed production of VEGF comparable to hIPod (Fig. 5A), a characteristic cortical rearrangement of actin filaments after PAN exposure (5 µg/ml for 5 days) (Fig. 5B–E), and formation of foot processes when all-trans retinoic acid (ATRA) is added to the differentiation medium (Fig. 2I). These characteristics were not found in the hFibroblasts (Fig. S5A–B).

Since in podocytes, intracellular calcium is an important second messenger involved in crucial cell signaling pathways [4], [23], we examined if the two cell lines increased their intracellular calcium concentration in response to flufenamic acid (FFA, a TRPC6 agonist) and angiotensin II (Ang II), using fluorescence imaging (Fig. 5F–J). The increase in intracellular calcium in hAKPC-P in response to FFA was significantly greater than in hIPod; hAKPC-P also exhibited intense contraction after FFA stimulation as shown in the Supplementary Video. Cells also showed an increase in intracellular calcium (Fig. 5J), contraction with a loss of cell protrusions and a decrease in apparent cytoplasmic area (Fig. 5K) in response to Ang II. After differentiation, hAKPC-P had higher expression of mRNA compared with re-differentiated hIPod for the calcium channel *TRPC6* (Fig. S5C–D), consistent with their stronger response to FFA. Differences in cytoskeletal proteins between differentiated hAKPC-P and re-differentiated hIPod are discussed in Supplementary Data (Fig. S6A–D and Supplementary Discussion S1).

### Cell Cycle Regulation in Differentiated hAKPC-P

**Figure 6:**
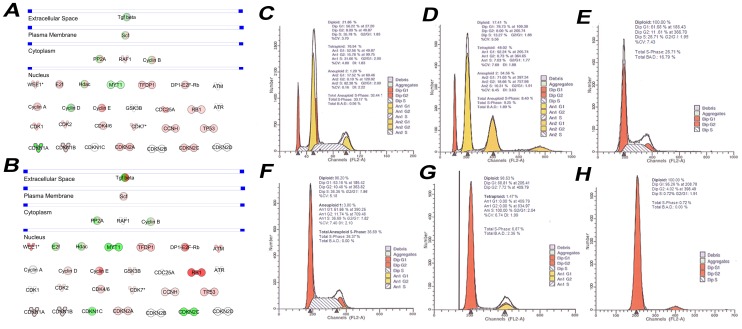
Cell cycle analysis of hAKPC-P and hIPod by microarray and FACS. A–B Ingenuity Pathways Analysis (IPA) of microarray data was used to identify significant differences in expression of genes fundamental to cell cycle regulation between undifferentiated hAKPC-P and de-differentiated hIPod (**A**, Table S4 in File S1) and between differentiated hAKPC-P and re-differentiated hIPod (**B**, Table S4 in File S1). Red symbols signify a higher mRNA content of hIPod, while green symbols signify a higher mRNA content in the hAKPC-P. Only significant differences (P<0.05) in gene expression are reported. Symbols with the same shape (oval, circle, diamond, etc.) share a similar function. **C**. Representative FACS cell cycle analysis of undifferentiated hIPod, in which aneuploid (1.2%), diploid (2n, 22%) and tetraploid (4n, 77%) cell populations were identified. In particular, an average of 33% of the cells (aneuploid, diploid and tetraploid) among all the populations was found in S-phase. **D**. Upon differentiation of hIPod, total S-phase decreased to 9% of the cells. Most cells still were not diploid. **E**. When de-differentiated again under permissive conditions (33°C) for 15 days, hIPod exhibited a purely diploid (2n) phenotype with about 27% of the cells in S-phase. **F**. The cell population of undifferentiated hAKPC-P was almost entirely diploid (2n, 96%) with a small percentage of cells exhibiting aneuploidy. About 36% of the cells (diploid plus aneuploid) were found in S-phase. **G**. After differentiation, most of the cells were still diploid (98%) with a small percentage of tetraploid (4n) cells. About 7% of the cells (diploid plus tetraploid) were in S-phase. **H**. After de-differentiation of hAKPC-P (by switching the culture to a medium without differentiative growth factors for 15 days), hAKPC-P exhibited only a diploid phenotype with less than 1% of the cells in S-phase.

Using microarray analysis we confirmed that differentiated hAKPC-P have an expression pattern of cell cycle proteins resembling *in vivo* podocytes. After differentiation (Fig. 6B), hAKPC-P had lower levels of mRNA for cyclins D and E, less CDK2 and CDK4/6, and increased levels of p57 (CDKN1C). Both populations exited their proliferative states (S phase) and maintained relative cell cycle arrest after differentiation (Fig. 6C–G). FACS cell cycle analysis in our study (Fig. 6D,G) revealed that hAKPC-P were much more often diploid than hIPod. When cells were switched back to either a permissive (for hIPod) or non-differentiative (hAKPC-P) culture system, after 14 days the polyploidy of the cells disappeared and was replaced by diploid-only populations (Fig. 6E–H).

### hAKPC-P Contains a Rare Population of Six-2^+^Cited1^+^ Cells

**Figure 7:**
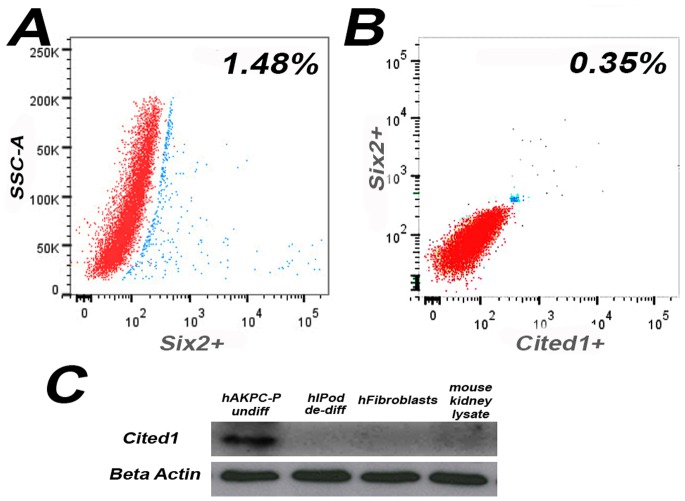
FACS analysis for early renal markers in undifferentiated hAKPC-P. FACS analysis showing the percentage of cells positive for Six2^+^ (**A**, 1.48%), Six2^+^Cited1^+^ (**B**, 0.35%) in undifferentiated hAKPC-P (Red line = unstained sample; Blue line = stained sample). **C**. Western blotting for Cited1 (20 kDa) in undifferentiated hAKPC-P, de-differentiated hIPod, hFibroblasts and mouse kidney lysate. While this protein, usually expressed during embryonic development, was not detected in hIPod, hFibroblasts and mouse kidney lysate, its expression in undifferentiated hAKPC-P suggests the stable presence of a subpopulation of self-renewing progenitors within hAKPC-P (housekeeping gene: beta-actin).

Prior to differentiation, flow cytometry on hAKPC-P confirmed the presence of cells positive for Six2 (1.48%), Cited1 (0.89%) and a subpopulation of cells double positive for Six2 and Cited1 (0.35%), (Fig. 7A–B). Expression of Cited1 protein in undifferentiated hAKPC-P, but not de-differentiated hIPod, hFibroblasts and mouse kidney lysate, was also confirmed by western blotting (Fig. 7C).

## Discussion

Stem cells, including renal endogenous stem cells, embryonic stem cells and also induced pluripotent stem cells hold promise for studying basic cell biology as well as for developing kidney cell-based therapies due to their capacity of *in vitro* proliferation and differentiation [24].

The number of studies reporting the differentiation of stem cells into podocytes, both *in vivo* and *in vitro*, is quite limited. In fact, only a few studies have described the induction of podocyte traits in stem cells, mainly using endogenous kidney progenitors, already committed to a renal fate [25]–[29]. The only work showing a typical functional characteristic of podocytes so derived has been recently reported by Song and colleagues, who, using iPS from a renal source of cells, showed the ability of differentiated cells to express characteristic podocyte proteins and to respond to stimulation by angiotensin II [30]. No publications to date have demonstrated the possibility of isolating, expanding and differentiating functional podocytes from an extra-renal source without the use of genetic manipulation.

We have previously demonstrated that amniotic fluid contains different types of organ progenitor cells, in addition to uncommitted stem cells selected by c-kit expression [12], [13].

Several fetal organs are in direct continuity with the amniotic fluid in the developing organism and we speculate that, given their route of entry into the amniotic fluid, hAKPC-P might represent a population of kidney epithelial cell progenitors which became detached from the glomerular tuft before undergoing terminal differentiation and transited via the tubule to the fetal bladder and thence into the amniotic fluid. We decided to use as selection markers for hAKPC-P, CD24 and OB-cadherin, in the first instance, because we have previously shown that these two markers are present in the amniotic fluid between the 16th and 18th week of gestation and that they identify a population of renal progenitors [12]. Both markers are strongly and specifically expressed *in vivo* in the uninduced metanephric mesenchyme (the embryonic tissue from which the entire kidney originates) and indicate the renal progenitor population [31]. Secondly we hypothesize that a more specific podocyte marker, like podocalyxin would represent the best marker to possibly sub-isolate podocyte progenitors [4]. OB-cadherin, CD24 and podocalyxin are expressed individually in other cell types; in fact, for example, hIPod and hFibroblasts express these markers singularly, but they were negative for the co-expression of the three markers. Thus, we speculate that this population selected from amniotic fluid represents the first exogenous stem-like cell that can be propagated *in vitro* and induced by culture conditions to differentiate into a podocyte [10].

In this work we compared *in vitro* hIPod with hAKPC-P. After differentiation, hAKPC-P acquire all the characteristics of cultured podocytes [4], [10]. In particular, they formed secondary processes with the approximate dimensions of foot processes [17] and formed contacts with neighboring cells at which major slit diaphragm proteins (ZO-1, podocin, nephrin) were expressed. Higher expression of podocyte-specific slit diaphragm specific proteins in hAKPC-P may underlie the greater frequency of cell-cell contacts found *in vitro* between these cells compared to hIPod.

With respect to the interaction of podocytes with their underlying GBM [4], we were able to identify the production of laminin α5 and, for the first time, of mature type IV collagen alpha chains from both hIPod and hAKPC-P. Microarray analysis suggests that hIPod may in addition produce matrix components not specific for podocytes, including fibronectin and other laminin chains (like β1). Podocyte-GBM interactions are mediated in part by two integrin dimers, principally α3β1, but also α1β1 [4]. Outside-in signaling to podocytes from integrin β1-matrix interactions is dependent on an integrin-linked kinase (ILK) [32]. Expression of proteins of all three mature type IV collagen chains (α3, α4, α5), of α3 and β1 integrins, and a developmental pattern of ILK expression suggest that hAKPC-P could represent a more suitable model for *in vitro* studies of glomerular development and cell-matrix interactions than traditional immortalized lines. After differentiation, hAKPC-P expressed not only these and important slit diaphragm proteins, but secreted VEGF, showed a typical cytoskeletal re-arrangement after exposure to puromycin aminonucleoside (PAN), formed foot processes in response to all-trans retinoic acid (ATRA) [10], and showed a high expression of TRPC6 mRNA and a pronounced Ca^2+^-influx in response to the TRPC6-agonist FFA, all defining characteristics of functional podocytes. In addition, hAKPC-P showed a distinct developmental pattern of expression for smoothelin mRNA and tropomyosin protein, two important cytoskeleton filament components involved in podocyte cell contraction through interaction with actin filaments [33], [34].

When podocytes are lost, there is little possibility to replace them from existing podocytes, as these are functionally post-mitotic cells [35]. The very low proliferative capacity of mature podocytes *in vivo* is largely due to the activity of cyclin-dependent kinase inhibitors CKI [36]. Podocytes stop dividing at the capillary loop stage of development, in association with upregulation of CKI, particularly p27 and p57 [37], and downregulation of cyclins A, B and E. This quiescent state corresponds to the initial expression of important markers of mature podocytes such as nephrin, WT1, and synaptopodin. Thus, cell cycle arrest seems to be a requirement for differentiated podocyte function. Podocytes have been shown to generate binucleated and multinucleated cells both *in vitro* and *in vivo* [4]. FACS cell cycle analysis in our studies revealed that hAKPC-P were much more often diploid than hIPod. Polyploidy has been found in hepatocytes during liver regeneration [38] and has been associated with a possible increased resistance to exogenous stress. Comparison of the mitotic behavior of the immortalized hIPod cell line with hAKPC-P might offer insights into the role of polyploidy in the podocyte’s response to cell stress *in situ*.

During embryonic development, podocytes originate within the cap mesenchyme (CM), a specific region of the metanephric mesenchyme that borders the ureteric tip during the formation of the renal vesicle [18], [39]. Some cells of the CM have self-renewal properties, while others proceed toward epithelialization and give rise to podocytes and other cells of the nephron. In particular, it has been shown that cells within the CM expressing Six2 and Cited1 undergo self-renewal during nephrogenesis and are involved in metanephric mesenchyme induction [10], [18], [39]–[41] but when stimulated, for example by Wnt4 or LIF, they can undergo epithelial differentiation [18]. Our findings show that hAKPC-P not only express generic early renal markers (OB-cadherin, CD24), but that some of the cells also express Six2 and Cited1. Thus, hAKPC-P, or at least a subpopulation within hAKPC-P, appear to be more than just direct podocyte progenitors, suggesting that hAKPC-P represent a mixed population of self-renewing cells committed to nephron specification and a population committed to podocyte differentiation. Immortalized podocytes, on the other hand, are mature podocytes that have been transformed by insertion of a temperature-sensitive mutant of the proto-oncogene, SV40 large T antigen, so that they de-differentiate and replicate under permissive conditions, and regain a podocyte phenotype under non-permissive conditions. They seem to lack the developmental range of hAKPC-P.

The inadequacy of available (immortalized) *in vitro* systems in this regard has been recently highlighted by Warsow and colleagues [42]. In contrast, hAKPC-P cells offer a promising and novel source of cultured podocytes that can be easily isolated from the amniotic fluid of humans or mammalian model organisms and do not require tedious genetic manipulation for immortalization in order to yield a useable *in vitro* system. Their characteristic developmental pattern of expression of several podocyte proteins suggests that, in addition to studying basic podocyte cell biology, hAKPC-P could represent the first *in vitro* model that can be used for analysis of podocyte development. Amniotic fluid-derived hAKPC-P thus offer the opportunity to explore the missing, elusive link between the metanephric mesenchyme progenitor cell and the fully mature podocyte. In this and other respects, they provide important insights not possible using available *in vitro* podocyte culture systems.

## Supporting Information

Figure S1 **FACS analysis for co-expression of podocyte progenitor markers CD24, OB-Cadherin and podocalyxin in mKC, hBM-MSCs, hAKPC-P differentiated and hIPod re-differentiated.** A–L. FACS analysis for CD24, OB-Cadherin and podocalyxin for mKC (**A–C**, 0.02% triple positive cells), hBM-MSCs (**D–E,** no triple positive cells), hAKPC-P after differentiation (**G–I,** 0.01% triple positive cells) and hIPod after re-differentiation (**J–L,** 1.77% triple positive cells). (Red line  =  unstained sample; Blue line  =  stained sample).
(TIF)

Figure S2 **FACS analysis for variation of podocyte progenitor markers CD24, OB-Cadherin and Podocalyxin in the different cell populations.** FACS analysis for CD24 (**A,D,G,J,M,P**), OB-Cadherin (**B,E,H,K,N,Q**) and podocalyxin (**C,F,I,L,O,R**). **A–C.** hAKPC-P differentiated showed that about 30% of the cells retained expression of CD24 **(A),** 5.75% expressed OB-Cadherin **(B)** and 27.8% maintained expression of podocalyxin **(C). D–F.** hIPod dedifferentiated showed that about 97.8% of the cells retained expression of CD24 **(D),** 26.1% expressed OB-Cadherin **(E)** and 4.48% were positive for podocalyxin **(F). G–I.** hIPod re-differentiated showed that about 89.9% of the cells retained expression of CD24 **(G),** 3.25% expressed OB-Cadherin **(H)** and 24.9% maintained expression of podocalyxin **(I). J–L.** About 21.5% % of the hFibroblasts were positive for CD24 **(J),** 60% expressed OB-Cadherin **(K)** and 1.54% showed expression of podocalyxin **(L). M–O.** About 0.44% of the hBM-MSCs were positive for CD24 **(M),** 0.13% expressed OB-Cadherin **(N)** and 0.16% showed expression of podocalyxin **(O). P–R.** About 5.89% % of the mKC cells were positive for CD24 **(P),** 1.67% expressed OB-Cadherin **(Q)** and 0.59% showed expression of podocalyxin **(R).** (Red line  =  unstained sample; Blue line  =  stained sample).
(TIF)

Figure S3 **Analysis of expression of specific podocyte markers, slit diaphragm protein expression and adherens-type junctions for undifferentiated hAKPC-P, de-differentiated hIPod, re-differentiated hIPod and hFibroblasts.** A–D. Representative pictures depicting immunofluorescence stainings for nephrin **(A),** podocin **(B),** ZO-1 **(C)** and CD151 (**D**) in undifferentiated hAKPC-P. **E–H**. De-differentiated hIPod showed expression for nephrin **(E)** while showing only faint expression of podocin **(F).** However, localization of podocin was not at cell-cell contacts. De-differentiated hIPod were also negative for ZO-1 (**G**) and CD151 (**H**). **I–L.** Upon re-differentiation hIPod expressed the slit diaphragm protein, nephrin. Unlike Fig. 2A, areas of cell-cell contacts do not express nephrin as in hAKPC-P (**I,** arrow pointing at cell-cell contact). Re-differentiated hIPod express podocin **(J)** and ZO-1. **(K).** Re**-**differentiated hIPod also showed expression of CD151 (**L**). **M–P.** hFibroblasts were negative for nephrin **(M),** podocin **(N),** ZO-1 **(O)** and CD151 (**P**). **Q–Y.** Before differentiation both hAKPC-P and hIPod were positive for WT1 and podocalyxin (**R,S,U,V**) and negative for synaptopodin (**Q,T**), while hFibroblasts were negative for all thee markers (**W,X,Y**). All pictures were taken at magnification equal to 40X with the exclusion of WT1, taken at 100X.
(TIF)

Figure S4 **Western Blotting Analysis of human fibroblasts and mouse kidney cortex for podocyte specific markers and collagen IV alpha chains.** A–B. Western blotting analysis of hFibroblasts and mouse kidney lysate for podocalyxin (160 kDa), podocin (42 kDa), and WT1 (51 kDa) and collagen IV alpha chains 1-2-3-4-5. Expression of both specific protein markers (**A**) and collagen IV alpha chains (25,50 kDa, **B**) was negative in hFibroblasts, but positive in the mouse kidney lysate.
(TIF)

Figure S5 **Cytoskeleton rearrangement in fibroblasts following PAN exposure and microarray analysis of calcium signaling specific genes.** A–B. Upon exposure to nephrotoxic agent puromycin aminonucleoside, hFibroblasts underwent apoptosis. However changes in actin cytoskeleton structure **(B,** arrows**)** compared to hFibroblast control **(A)** did not show the characteristic cortical rearrangement seen in both hIPod and hAKPC-P. **C–D.** Ingenuity Pathways Analysis (IPA) of microarray data was used to identify significant differences in expression of genes involved in Ca^++^ signaling between undifferentiated hAKPC-P and dedifferentiated hIPod (**C**) and between differentiated hAKPC-P and re-differentiated hIPod (**D**) (Table S5 in File S1). Red symbols signify a higher mRNA content in re-differentiated hIPod, while green symbols signify a higher mRNA content in differentiated hAKPC-P. Only significant differences (P<0.05) in gene expression are reported. Symbols with the same shape (oval, circle, diamond, etc.) share a similar function.
(TIF)

Figure S6 **Analysis of undifferentiated and differentiated hAKPC-P and hIPod for contractility markers.** **A–B.** Ingenuity Pathways Analysis (IPA) of microarray data was used to identify significant differences in expression of genes involved in contractility between undifferentiated hAKPC-P and de-differentiated hIPod (**A,** Table S6), and differentiated hAKPC-P and re-differentiated hIPod (**B,** Table S6 in File S1). Red symbols signify a higher mRNA content of hIPod, while green symbols signify a higher mRNA content in the hAKPC-P. Only significant differences (P<0.05) in gene expression are reported. Symbols with the same shape (oval, circle, diamond, etc.) share a similar function. **C.** After differentiation, hAKPC-P started expressing smoothelin as shown by quantitative real time PCR analysis performed using standard protocols [13] (Forward: aggtggccttctcatctgc; Reverse: ccgcaccatgtcctctgta; Probe from Roche Universal Probe Library: 17). **D.** Western blot analysis showing a large increase in tropomyosin protein (55 kDa) expression occurred in hAKPC-P after differentiation, whereas levels of protein expression did not change between undifferentiated and differentiated hIPod (housekeeping gene: beta-actin).
(TIF)

Video S1 **Representative video of hAKPC-P during FFA administration. DIC overlay of Fluo-4 (green) and Fura Red (red) is shown. Intense cell contractions occur simultaneously with the increases in [Ca^2+^]_i_.**
(MOV)

Discussion S1 **Contraction in hAKPC-P and hIPod. Supplementary discussion on the comparison of gene and protein expression of contractility markers in hAKPC-P and hIPod.**
(PDF)

File S1 **List of antibodies, catalogue numbers and concentrations used for various assays including immunofluorescence, FACS analysis and western blot analysis (Table S1) and list of all fold change values for the significant (P<0.05) microarray data presented in the main figures (Tables S2–S6).** Table S1 – List of antibodies used for immunofluorescence, flow cytometry and western blot analysis. Antibodies used in this study are listed along with catalogue numbers and concentration and whenever applicable, protein size expressed in kDa. IF = Immunofluorescence, WB = Western Blotting, FC = Flow Cytometry. Table S2 – Summary table reporting fold variation for podocyte and slit diaphragm markers A detailed list of podocyte and slit diaphragm genes compared for differential expression between de-differentiated hIPod and undifferentiated hAKPC-P (on the left, light grey) and between re-differentiated hIPod and differentiated hAKPC-P (on the right, dark grey). Positive values indicate a higher gene expression in the hIPod versus the hAKPC-P, while negative values indicate a higher gene expression in hAKPC-P. All fold variations listed are statistically significant (P<0.05). Table S3 – Summary table reporting fold variation for markers for GBM formation A detailed list of GBM related genes compared for differential expression between de-differentiated hIPod and undifferentiated hAKPC-P (on the left, light grey) and between re-differentiated hIPod and differentiated hAKPC-P (on the right, dark grey). Positive values indicate a higher gene expression in the hIPod versus the hAKPC-P, while negative values indicate a higher gene expression in hAKPC-P. All fold variations listed are statistically significant (P<0.05). Table S4 – Summary table reporting fold variation for markers involved in cell cycle regulation in podocytes A detailed list of cell cycle related genes compared for differential expression between de-differentiated hIPod and undifferentiated hAKPC-P (on the left, light grey) and between re-differentiated hIPod and differentiated hAKPC-P (on the right, dark grey). Positive values indicate a higher gene expression in the hIPod versus the hAKPC-P, while negative values indicate a higher gene expression in hAKPC-P. All fold variations listed are statistically significant (P<0.05). Table S5 – Summary table reporting fold variation for markers involved in Ca++ signaling in podocytes A detailed list of Ca++ signaling related genes compared for differential expression between de-differentiated hIPod and undifferentiated hAKPC-P (on the left, light grey) and between re-differentiated hIPod and differentiated hAKPC-P (on the right, dark grey). Positive values indicate a higher gene expression in the hIPod versus the hAKPC-P, while negative values indicate a higher gene expression in hAKPC-P. All fold variations listed are statistically significant (P<0.05). Table S6 – Summary table reporting fold variation for contractility markers in podocytes A detailed list of contractility genes compared for differential expression between de-differentiated hIPod and undifferentiated hAKPC-P (on the left, light grey) and between re-differentiated hIPod and differentiated hAKPC-P (on the right, dark grey). Positive values indicate a higher gene expression in the hIPod versus the hAKPC-P, while negative values indicate a higher gene expression in hAKPC-P. All fold variations listed are statistically significant (P<0.05).
(PDF)

## References

[1] Kriz W, Gretz N, Lemley KV (1998) Progression of glomerular diseases: is the podocyte the culprit? Kidney Int 54: 687–697. 9734594 10.1046/j.1523-1755.1998.00044.x

[2] Pagtalunan ME, Miller PL, Jumping-Eagle S, Nelson RG, Myers BD, et al (1997) Podocyte loss and progressive glomerular injury in type II diabetes. J Clin Invest 99: 342–348. 9006003 10.1172/JCI119163 PMC507802

[3] Fukuda A, Wickman LT, Venkatareddy MP, Sato Y, Chowdhury MA, et al (2012) Angiotensin II-dependent persistent podocyte loss from destabilized glomeruli causes progression of end stage kidney disease. Kidney Int 81: 40–55. 21937979 10.1038/ki.2011.306 PMC3739490

[4] Pavenstadt H, Kriz W, Kretzler M (2003) Cell biology of the glomerular podocyte. Physiol Rev 83: 253–307. 12506131 10.1152/physrev.00020.2002

[5] Norgaard JO (1987) Rat glomerular epithelial cells in culture. Parietal or visceral epithelial origin? Lab Invest 57: 277–290. 3626518

[6] Mundel P, Kriz W (1996) Cell culture of podocytes. Exp Nephrol 4: 263–266. 8931980

[7] Saleem MA, O’Hare MJ, Reiser J, Coward RJ, Inward CD, et al (2002) A conditionally immortalized human podocyte cell line demonstrating nephrin and podocin expression. J Am Soc Nephrol 13: 630–638. 11856766 10.1681/ASN.V133630

[8] Ransom RF, Lam NG, Hallett MA, Atkinson SJ, Smoyer WE (2005) Glucocorticoids protect and enhance recovery of cultured murine podocytes via actin filament stabilization. Kidney Int 68: 2473–2483. 16316324 10.1111/j.1523-1755.2005.00723.x

[9] Faul C, Donnelly M, Merscher-Gomez S, Chang YH, Franz S, et al (2008) The actin cytoskeleton of kidney podocytes is a direct target of the antiproteinuric effect of cyclosporine A. Nat Med. 14: 931–938. 10.1038/nm.1857 PMC4109287 18724379

[10] Shankland SJ, Pippin JW, Reiser J, Mundel P (2007) Podocytes in culture: past, present, and future. Kidney Int 72: 26–36. 17457377 10.1038/sj.ki.5002291

[11] Chittiprol S, Chen P, Petrovic-Djergovic D, Eichler T, Ransom RF (2011) Marker expression, behaviors, and responses vary in different lines of conditionally immortalized cultured podocytes. Am J Physiol Renal Physiol 301: F660–671. 21632959 10.1152/ajprenal.00234.2011 PMC3174553

[12] Da Sacco S, Sedrakyan S, Boldrin F, Giuliani S, Parnigotto P, et al (2010) Human amniotic fluid as a potential new source of organ specific precursor cells for future regenerative medicine applications. J Urol 183: 1193–1200. 20096867 10.1016/j.juro.2009.11.006 PMC3174101

[13] Sedrakyan S, Da Sacco S, Milanesi A, Shiri L, Petrosyan A, et al (2012) Injection of amniotic fluid stem cells delays progression of renal fibrosis. J Am Soc Nephrol 23: 661–673. 22302195 10.1681/ASN.2011030243 PMC3312511

[14] Meezan E, Hjelle JT, Brendel K, Carlson EC (1975) A simple, versatile, nondisruptive method for the isolation of morphologically and chemically pure basement membranes from several tissues. Life Sci 17: 1721–1732. 1207385 10.1016/0024-3205(75)90119-8

[15] Takemoto M, Asker N, Gerhardt H, Lundkvist A, Johansson BR, et al (2002) A new method for large scale isolation of kidney glomeruli from mice. Am J Pathol 161: 799–805. 12213707 10.1016/S0002-9440(10)64239-3 PMC1867262

[16] Takano Y, Yamauchi K, Hiramatsu N, et al. Recovery and maintenance of nephrin expression in cultured podocytes and identification of HGF as a repressor of nephrin. American journal of physiology Renal physiology 292: F1573–1582. 10.1152/ajprenal.00423.2006 17244893

[17] Lemley KV, Lafayette RA, Safai M, Derby G, Blouch K, et al (2002) Podocytopenia and disease severity in IgA nephropathy. Kidney Int 61: 1475–1485. 11918755 10.1046/j.1523-1755.2002.00269.x

[18] Little MH, McMahon AP. Mammalian kidney development: principles, progress, and projections. Cold Spring Harbor perspectives in biology 2012; 4. 10.1101/cshperspect.a008300 PMC3331696 22550230

[19] Holthofer H (2007) Molecular architecture of the glomerular slit diaphragm: lessons learnt for a better understanding of disease pathogenesis. Nephrol Dial Transplant 22: 2124–2128. 17550922 10.1093/ndt/gfm344

[20] Reiser J, Kriz W, Kretzler M, Mundel P (2000) The glomerular slit diaphragm is a modified adherens junction. J Am Soc Nephrol 11: 1–8. 10616834 10.1681/ASN.V1111

[21] Schordan S, Schordan E, Endlich K, Endlich N (2011) AlphaV-integrins mediate the mechanoprotective action of osteopontin in podocytes. Am J Physiol Renal Physiol 300: F119–132. 21048023 10.1152/ajprenal.00143.2010

[22] Pozzi A, Zent R (2012) Hold tight or you’ll fall off: CD151 helps podocytes stick in high-pressure situations. J Clin Invest 122: 13–16. 22201676 10.1172/JCI61858 PMC3248315

[23] Greka A, Mundel P (2011) Balancing calcium signals through TRPC5 and TRPC6 in podocytes. J Am Soc Nephrol 22: 1969–1980. 21980113 10.1681/ASN.2011040370 PMC3231779

[24] Perin L, Da Sacco S, De Filippo RE (2011) Regenerative medicine of the kidney. Adv Drug Deliv Rev 63: 379–387. 21145933 10.1016/j.addr.2010.12.001

[25] Ronconi E, Sagrinati C, Angelotti ML, Lazzeri E, Mazzinghi B, et al (2009) Regeneration of glomerular podocytes by human renal progenitors. J Am Soc Nephrol 20: 322–332. 19092120 10.1681/ASN.2008070709 PMC2637058

[26] Kramer J, Steinhoff J, Klinger M, Fricke L, Rohwedel J (2006) Cells differentiated from mouse embryonic stem cells via embryoid bodies express renal marker molecules. Differentiation 74: 91–104. 16533308 10.1111/j.1432-0436.2006.00062.x

[27] Bruno S, Bussolati B, Grange C, Collino F, di Cantogno LV, et al (2009) Isolation and characterization of resident mesenchymal stem cells in human glomeruli. Stem Cells Dev 18: 867–880. 19579288 10.1089/scd.2008.0320

[28] Fuente Mora C, Ranghini E, Bruno S, Bussolati B, Camussi G, et al (2012) Differentiation of podocyte and proximal tubule-like cells from a mouse kidney-derived stem cell line. Stem Cells Dev 21: 296–307. 21510739 10.1089/scd.2010.0470

[29] Perry J, Tam S, Zheng K, Sado Y, Dobson H, et al (2006) Type IV collagen induces podocytic features in bone marrow stromal stem cells in vitro. J Am Soc Nephrol 17: 66–76. 16280470 10.1681/ASN.2005060586

[30] Song B, Smink AM, Jones CV, Callaghan JM, Firth SD, et al (2012) The directed differentiation of human iPS cells into kidney podocytes. PLoS One 7: e46453. 23029522 10.1371/journal.pone.0046453 PMC3460883

[31] Challen GA, Martinez G, Davis MJ, Taylor DF, Crowe M, et al (2004) Identifying the molecular phenotype of renal progenitor cells. J Am Soc Nephrol 15: 2344–2357. 15339983 10.1097/01.ASN.0000136779.17837.8F

[32] Dai C, Stolz DB, Bastacky SI, St-Arnaud R, Wu C, et al (2006) Essential role of integrin-linked kinase in podocyte biology: Bridging the integrin and slit diaphragm signaling. J Am Soc Nephrol 17: 2164–2175. 16837631 10.1681/ASN.2006010033

[33] Saleem MA, Zavadil J, Bailly M, McGee K, Witherden IR, et al (2008) The molecular and functional phenotype of glomerular podocytes reveals key features of contractile smooth muscle cells. Am J Physiol Renal Physiol 295: F959–970. 18684887 10.1152/ajprenal.00559.2007 PMC2576149

[34] Brunskill EW, Georgas K, Rumballe B, Little MH, Potter SS (2011) Defining the molecular character of the developing and adult kidney podocyte. PLoS One 6: e24640. 21931791 10.1371/journal.pone.0024640 PMC3169617

[35] Marshall CB, Shankland SJ (2006) Cell cycle and glomerular disease: a minireview. Nephron Exp Nephrol 102: e39–48. 16179806 10.1159/000088400

[36] Shankland SJ, Eitner F, Hudkins KL, Goodpaster T, D’Agati V, et al (2000) Differential expression of cyclin-dependent kinase inhibitors in human glomerular disease: role in podocyte proliferation and maturation. Kidney Int 58: 674–683. 10916090 10.1046/j.1523-1755.2000.00213.x

[37] Nagata M, Shibata S, Shigeta M, Yu-Ming S, Watanabe T (1999) Cyclin-dependent kinase inhibitors: p27kip1 and p57kip2 expression during human podocyte differentiation. Nephrol Dial Transplant 14 Suppl 148–51. 10.1093/ndt/14.suppl_1.48 10048450

[38] Duncan AW, Taylor MH, Hickey RD, et al. The ploidy conveyor of mature hepatocytes as a source of genetic variation. Nature 467: 707–710. 20861837 10.1038/nature09414 PMC2967727

[39] Hendry C, Rumballe B, Moritz K, Little MH (2011) Defining and redefining the nephron progenitor population. Pediatr Nephrol 26: 1395–1406. 21229268 10.1007/s00467-010-1750-4 PMC3189495

[40] Mugford JW, Yu J, Kobayashi A, McMahon AP (2009) High-resolution gene expression analysis of the developing mouse kidney defines novel cellular compartments within the nephron progenitor population. Dev Biol 333: 312–323. 19591821 10.1016/j.ydbio.2009.06.043 PMC2748313

[41] Quaggin SE, Kreidberg JA (2008) Development of the renal glomerulus: good neighbors and good fences. Development 135: 609–620. 18184729 10.1242/dev.001081

[42] Warsow G, Endlich N, Schordan E, Schordan S, Chilukoti RK, et al (2013) PodNet, a protein-protein interaction network of the podocyte. Kidney Int 84: 104–115. 23552858 10.1038/ki.2013.64

