# Supplementary figures and images for: A Novel Source of Cultured Podocytes
Source: PLoS One. 2013 Dec 12;8(12):e81812. doi: 10.1371/journal.pone.0081812 (PMC3861313; doi:10.1371/journal.pone.0081812)

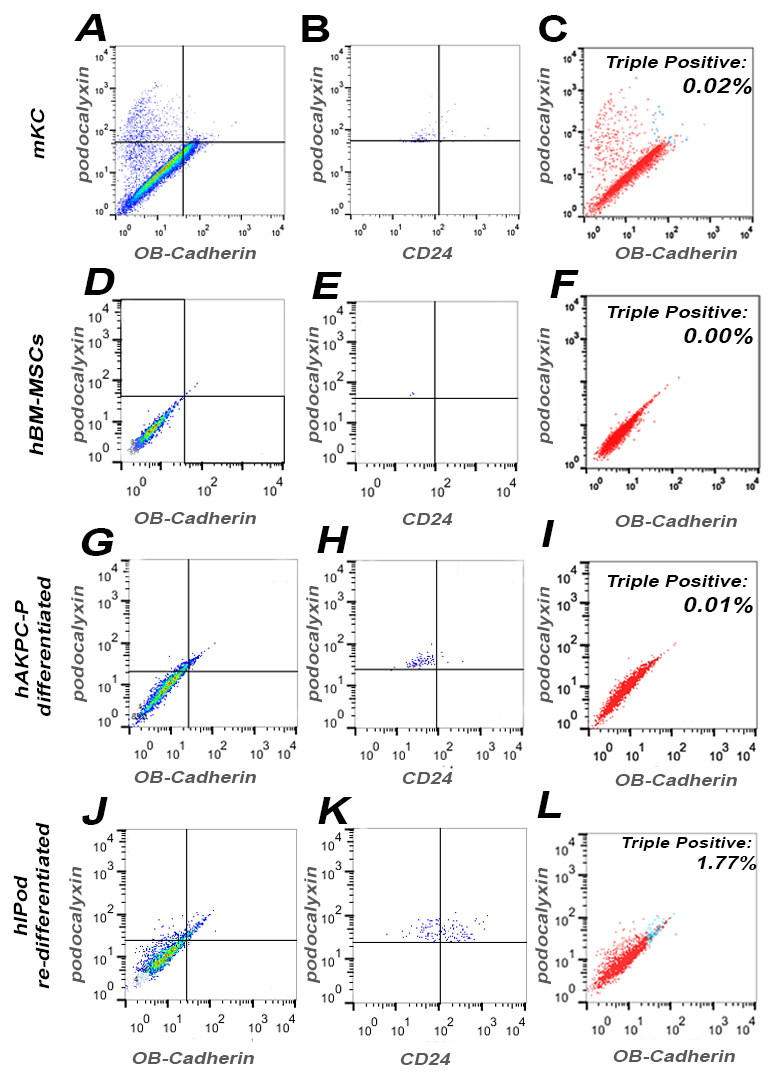

Supplement: Figure S1 — FACS analysis for co-expression of podocyte progenitor markers CD24, OB-Cadherin and podocalyxin in mKC, hBM-MSCs, hAKPC-P differentiated and hIPod re-differentiated. A–L. FACS analysis for CD24, OB-Cadherin and podocalyxin for mKC (A–C, 0.02% triple positive cells), hBM-MSCs (D–E, no triple positive cells), hAKPC-P after differentiation (G–I, 0.01% triple positive cells) and hIPod after re-differentiation (J–L, 1.77% triple positive cells). (Red line = unstained sample; Blue line = stained sample). (TIF) [file pone.0081812.s001.tif]

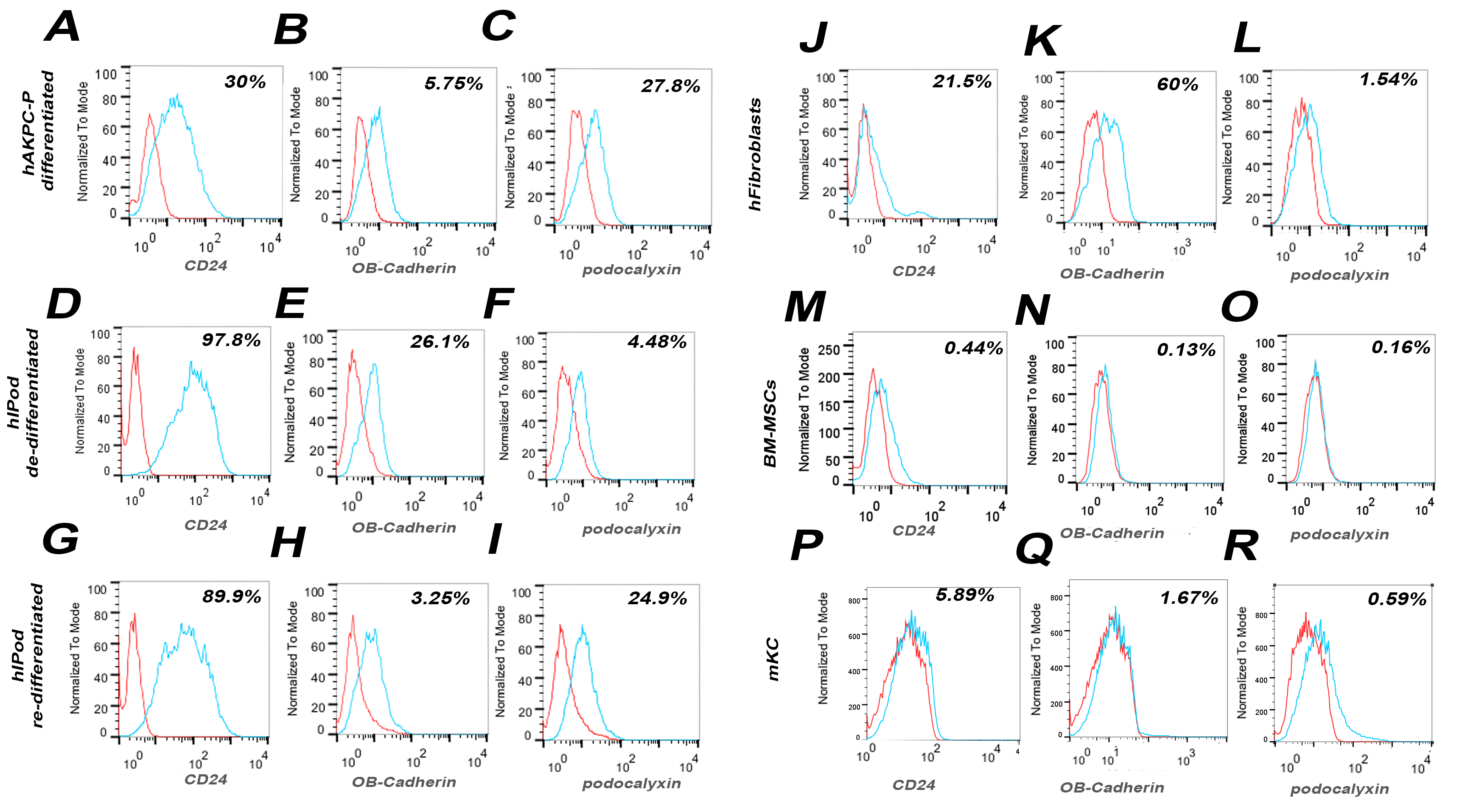

Supplement: Figure S2 — FACS analysis for variation of podocyte progenitor markers CD24, OB-Cadherin and Podocalyxin in the different cell populations. FACS analysis for CD24 (A,D,G,J,M,P), OB-Cadherin (B,E,H,K,N,Q) and podocalyxin (C,F,I,L,O,R). A–C. hAKPC-P differentiated showed that about 30% of the cells retained expression of CD24 (A), 5.75% expressed OB-Cadherin (B) and 27.8% maintained expression of podocalyxin (C). D–F. hIPod dedifferentiated showed that about 97.8% of the cells retained expression of CD24 (D), 26.1% expressed OB-Cadherin (E) and 4.48% were positive for podocalyxin (F). G–I. hIPod re-differentiated showed that about 89.9% of the cells retained expression of CD24 (G), 3.25% expressed OB-Cadherin (H) and 24.9% maintained expression of podocalyxin (I). J–L. About 21.5% % of the hFibroblasts were positive for CD24 (J), 60% expressed OB-Cadherin (K) and 1.54% showed expression of podocalyxin (L). M–O. About 0.44% of the hBM-MSCs were positive for CD24 (M), 0.13% expressed OB-Cadherin (N) and 0.16% showed expression of podocalyxin (O). P–R. About 5.89% % of the mKC cells were positive for CD24 (P), 1.67% expressed OB-Cadherin (Q) and 0.59% showed expression of podocalyxin (R). (Red line = unstained sample; Blue line = stained sample). (TIF) [file pone.0081812.s002.tif]

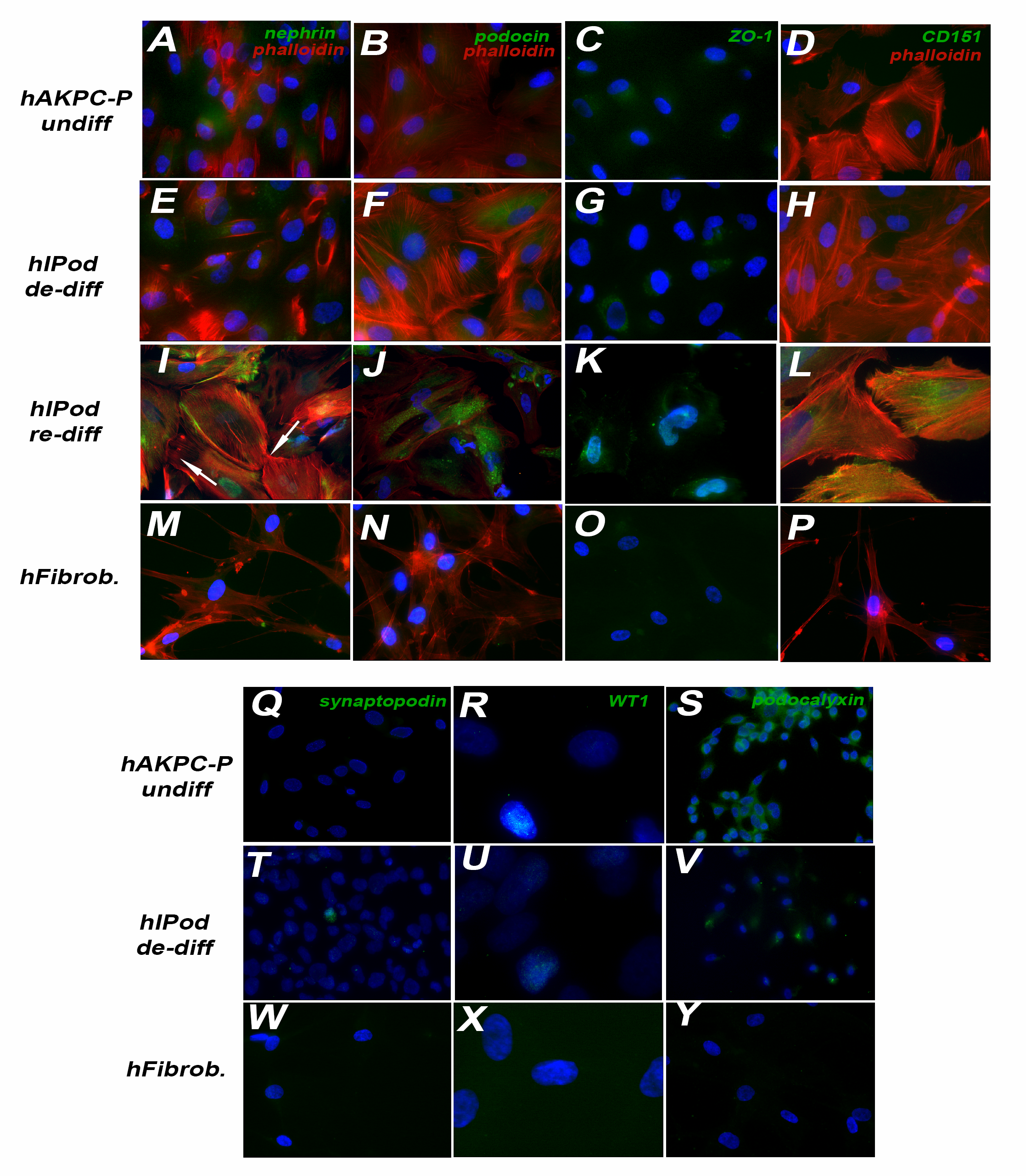

Supplement: Figure S3 — Analysis of expression of specific podocyte markers, slit diaphragm protein expression and adherens-type junctions for undifferentiated hAKPC-P, de-differentiated hIPod, re-differentiated hIPod and hFibroblasts. A–D. Representative pictures depicting immunofluorescence stainings for nephrin (A), podocin (B), ZO-1 (C) and CD151 (D) in undifferentiated hAKPC-P. E–H. De-differentiated hIPod showed expression for nephrin (E) while showing only faint expression of podocin (F). However, localization of podocin was not at cell-cell contacts. De-differentiated hIPod were also negative for ZO-1 (G) and CD151 (H). I–L. Upon re-differentiation hIPod expressed the slit diaphragm protein, nephrin. Unlike Fig. 2A, areas of cell-cell contacts do not express nephrin as in hAKPC-P (I, arrow pointing at cell-cell contact). Re-differentiated hIPod express podocin (J) and ZO-1. (K). Re-differentiated hIPod also showed expression of CD151 (L). M–P. hFibroblasts were negative for nephrin (M), podocin (N), ZO-1 (O) and CD151 (P). Q–Y. Before differentiation both hAKPC-P and hIPod were positive for WT1 and podocalyxin (R,S,U,V) and negative for synaptopodin (Q,T), while hFibroblasts were negative for all thee markers (W,X,Y). All pictures were taken at magnification equal to 40X with the exclusion of WT1, taken at 100X. (TIF) [file pone.0081812.s003.tif]

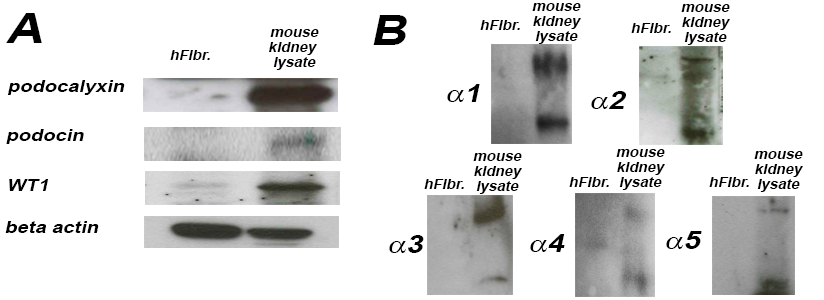

Supplement: Figure S4 — Western Blotting Analysis of human fibroblasts and mouse kidney cortex for podocyte specific markers and collagen IV alpha chains. A–B. Western blotting analysis of hFibroblasts and mouse kidney lysate for podocalyxin (160 kDa), podocin (42 kDa), and WT1 (51 kDa) and collagen IV alpha chains 1-2-3-4-5. Expression of both specific protein markers (A) and collagen IV alpha chains (25,50 kDa, B) was negative in hFibroblasts, but positive in the mouse kidney lysate. (TIF) [file pone.0081812.s004.tif]

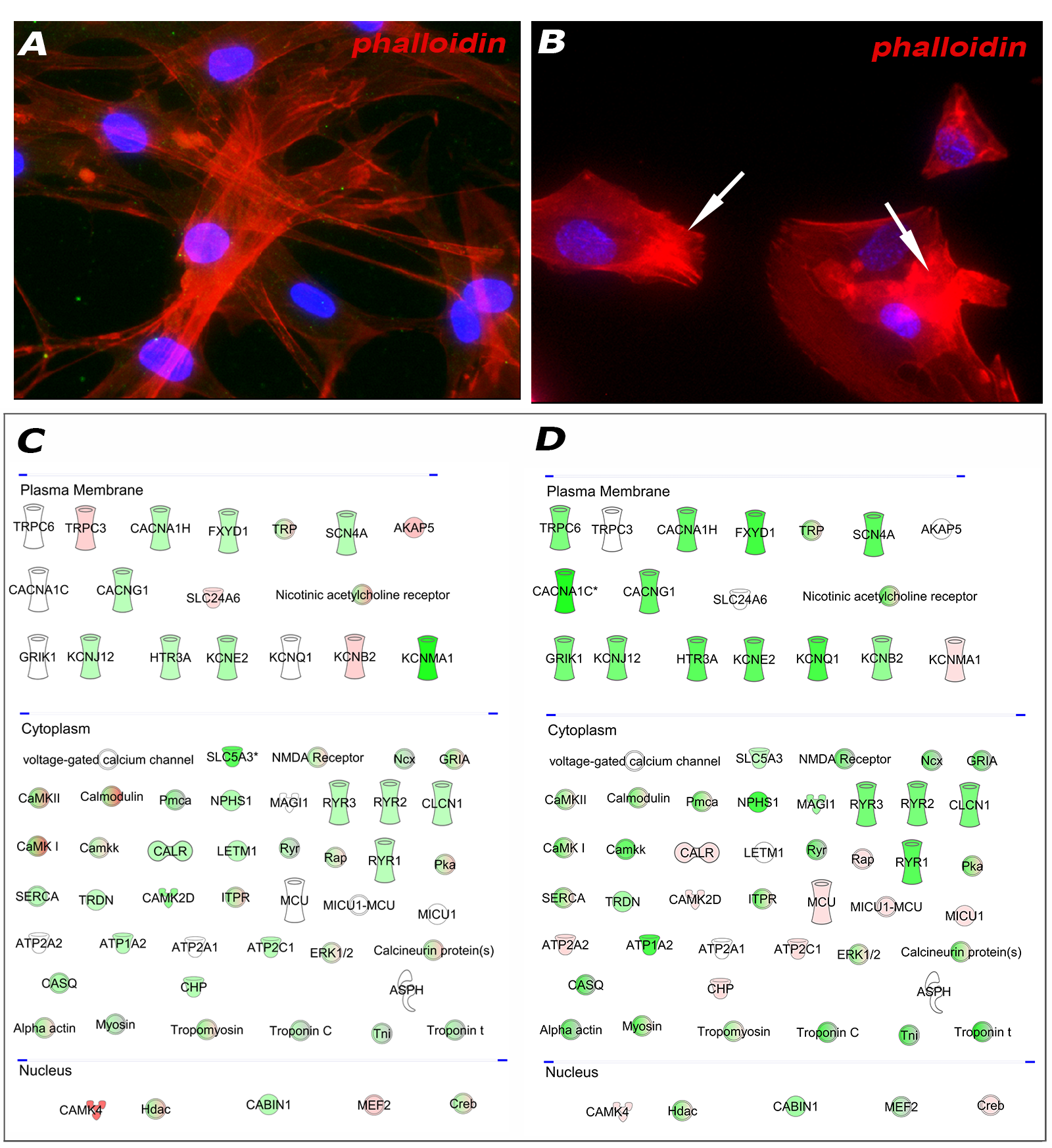

Supplement: Figure S5 — Cytoskeleton rearrangement in fibroblasts following PAN exposure and microarray analysis of calcium signaling specific genes. A–B. Upon exposure to nephrotoxic agent puromycin aminonucleoside, hFibroblasts underwent apoptosis. However changes in actin cytoskeleton structure (B, arrows) compared to hFibroblast control (A) did not show the characteristic cortical rearrangement seen in both hIPod and hAKPC-P. C–D. Ingenuity Pathways Analysis (IPA) of microarray data was used to identify significant differences in expression of genes involved in Ca++ signaling between undifferentiated hAKPC-P and dedifferentiated hIPod (C) and between differentiated hAKPC-P and re-differentiated hIPod (D) (Table S5 in File S1). Red symbols signify a higher mRNA content in re-differentiated hIPod, while green symbols signify a higher mRNA content in differentiated hAKPC-P. Only significant differences (P<0.05) in gene expression are reported. Symbols with the same shape (oval, circle, diamond, etc.) share a similar function. (TIF) [file pone.0081812.s005.tif]

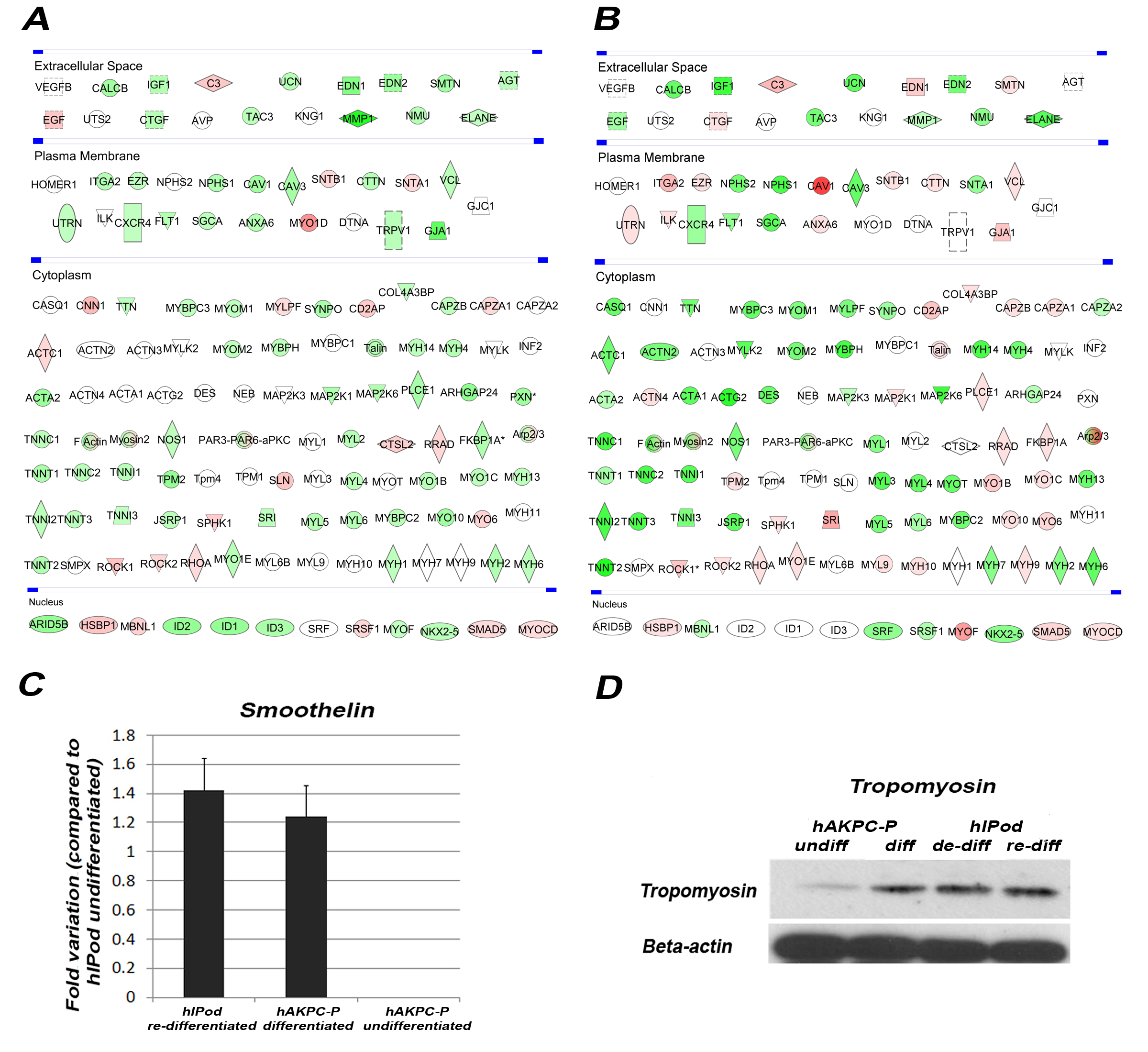

Supplement: Figure S6 — Analysis of undifferentiated and differentiated hAKPC-P and hIPod for contractility markers. A–B. Ingenuity Pathways Analysis (IPA) of microarray data was used to identify significant differences in expression of genes involved in contractility between undifferentiated hAKPC-P and de-differentiated hIPod (A, Table S6), and differentiated hAKPC-P and re-differentiated hIPod (B, Table S6 in File S1). Red symbols signify a higher mRNA content of hIPod, while green symbols signify a higher mRNA content in the hAKPC-P. Only significant differences (P<0.05) in gene expression are reported. Symbols with the same shape (oval, circle, diamond, etc.) share a similar function. C. After differentiation, hAKPC-P started expressing smoothelin as shown by quantitative real time PCR analysis performed using standard protocols [13] (Forward: aggtggccttctcatctgc; Reverse: ccgcaccatgtcctctgta; Probe from Roche Universal Probe Library: 17). D. Western blot analysis showing a large increase in tropomyosin protein (55 kDa) expression occurred in hAKPC-P after differentiation, whereas levels of protein expression did not change between undifferentiated and differentiated hIPod (housekeeping gene: beta-actin). (TIF) [file pone.0081812.s006.tif]
