# Supplementary material for: A Novel Source of Cultured Podocytes
Source: PLoS One. 2013 Dec 12;8(12):e81812. doi: 10.1371/journal.pone.0081812 (PMC3861313; doi:10.1371/journal.pone.0081812)
Supplement: Discussion S1 — Contraction in hAKPC-P and hIPod. Supplementary discussion on the comparison of gene and protein expression of contractility markers in hAKPC-P and hIPod. (PDF) [file pone.0081812.s008.pdf]

Supplementary Discussion:

The complex morphology of the podocyte depends on its highly organized cytoskeleton [1]. The podocyte is both a contractile cell and also a motile cell and its function therefore depends on a collection of actin- and myosin-associated proteins [2] (actin, α-actinin-4, other actin related proteins like Arp 2/3 complex and inverted formin 2 (INF2); non-muscle myosin light and heavy chains and myosin II). Podocyte cell polarity depends on the Par3/Par6/aPKC complex [3]. Ca2+ signaling (in particular via TRPC5 and TRPC6 channels) is substantially involved in the maintenance of the cytoskeleton organization in podocytes [4]. At the cytoplasmic level, differentiated hAKPC-P had higher expression levels of mRNA for troponins, actin 1 and 2 and actinin 2, while hIPod expressed more actinin-4 message (Supplementary Figure S6B). Differentiated hAKPC-P had higher levels of message for synaptopodin, desmin and myosin II and for the complex Par3/Par6/aPKC. mRNA levels for non-muscle myosin heavy and light chains are highly expressed in differentiated hAKPC-P, but *MYL9*, identified in cultured podocytes [2], is expressed at slightly higher levels in re-differentiated hIPod.

Supplementary Bibliography

1. Faul C, Donnelly M, Merscher-Gomez S, Chang YH, Franz S, et al. (2008) The actin cytoskeleton of kidney podocytes is a direct target of the antiproteinuric effect of cyclosporine A. Nat Med 14: 931-938.
2. Noris, M., Remuzzi, G. (2012) Non-muscle myosins and the podocyte. Clin Kidney J **5** (2): 94-101.

3. Huber, T. B, Hartleben B, Winkelmann K, Schneider L., Becker JU et al. (2009) Loss of podocyte aPKClambda/iota causes polarity defects and nephrotic syndrome. Journal of the American Society of Nephrology **20**, 798-806 .
4. Greka, A. & Mundel, P. (2011) Balancing calcium signals through TRPC5 and TRPC6 in podocytes. Journal of the American Society of Nephrology **22**, 1969-1980.
