# Supplementary material for: A Novel Source of Cultured Podocytes
Source: PLoS One. 2013 Dec 12;8(12):e81812. doi: 10.1371/journal.pone.0081812 (PMC3861313; doi:10.1371/journal.pone.0081812)
Supplement: File S1 — List of antibodies, catalogue numbers and concentrations used for various assays including immunofluorescence, FACS analysis and western blot analysis (Table S1) and list of all fold change values for the significant (P<0.05) microarray data presented in the main figures (Tables S2–S6). Table S1 – List of antibodies used for immunofluorescence, flow cytometry and western blot analysis. Antibodies used in this study are listed along with catalogue numbers and concentration and whenever applicable, protein size expressed in kDa. IF = Immunofluorescence, WB = Western Blotting, FC = Flow Cytometry. Table S2 – Summary table reporting fold variation for podocyte and slit diaphragm markers A detailed list of podocyte and slit diaphragm genes compared for differential expression between de-differentiated hIPod and undifferentiated hAKPC-P (on the left, light grey) and between re-differentiated hIPod and differentiated hAKPC-P (on the right, dark grey). Positive values indicate a higher gene expression in the hIPod versus the hAKPC-P, while negative values indicate a higher gene expression in hAKPC-P. All fold variations listed are statistically significant (P<0.05). Table S3 – Summary table reporting fold variation for markers for GBM formation A detailed list of GBM related genes compared for differential expression between de-differentiated hIPod and undifferentiated hAKPC-P (on the left, light grey) and between re-differentiated hIPod and differentiated hAKPC-P (on the right, dark grey). Positive values indicate a higher gene expression in the hIPod versus the hAKPC-P, while negative values indicate a higher gene expression in hAKPC-P. All fold variations listed are statistically significant (P<0.05). Table S4 – Summary table reporting fold variation for markers involved in cell cycle regulation in podocytes A detailed list of cell cycle related genes compared for differential expression between de-differentiated hIPod and undifferentiated hAKPC-P (on the left, light gre [file pone.0081812.s009.pdf]

Table S1 - List of antibodies used for immunofluorescence, flow cytometry and western blot analysis. Antibodies used in this study are listed along with catalogue numbers and concentration and whenever applicable, protein size expressed in kDa. IF = Immunofluorescence, WB = Western Blotting, FC = Flow Cytometry

PROTEIN	Antibody	Concentration	Band Size (Kda)
**Actin Beta**	GeneTex #109639	1:10000	42 kDa
**CD151**	Epitomics #5901-1	1:100 (IF)	----
**CITED1**	Abcam #ab55467	5mg/ml (WB)	20 kDa
	Novus Biological #H00004435-M03	1mg/ml (FC)	----
**COL4A1**	Shigei Research Institute #H12	1:500 (WB)	25, 50 kDa
**COL4A2**	Shigei Research Institute #H22	1:500 (WB)	25, 50 kDa
**COL4A3**	Shigei Research Institute #H31	1:500 (WB)	25, 50 kDa
**COL4A4**	Shigei Research Institute #RH42	1:500 (WB)	25, 50 kDa
**COL4A5**	Shigei Research Institute #H53	1:500 (WB)	25, 50 kDa
**ILK**	Abcam #ab52480	1:500 (WB)	51 Kda
**Integrin alpha 1**	Abcam #ab106267	0.8mg/ml (WB)	131 kDa
**Integrin alpha 3**	Abcam #ab251428	3.3mg/ml (WB)	131 kDa
**Integrin beta 5**	Abcam #ab95623	2.5mg/ml (WB)	88 kDa
**Laminin alpha 5**	Abbiotec #251457	2mg/ml (WB)	37kDa
**Nephrin**	Santa Cruz #sc-28192	1mg/ml (IF)	----
	Thermo Scientific PA5-25932	1mg/ml (FC)	----
**P-Cadherin**	Cell Signaling, #2189	1:25 (IF)	----
**Phalloidin**	Invitrogen #R415	1:1000 (IF)	----
**Podocalyxin**	Invitrogen #393800	1:100 (IF)	----
		1mg/ml (WB)	160 kDa
	R&D #MAB1556	1mg/ml (FC mouse)	----
**Podocin**	Abcam #ab50339	1:100 (IF)	----
		1mg/ml (WB)	42 kDa
**Six-2**	Santa Cruz	1mg/ml (FC)	----
**Synaptopodin**	USBio #S9500	1:100 (IF)	----
**Tropomyosin**	Abcam #7785	8mg/ml	55 kDa
**VEGF**	Abcam #ab46154	1mg/ml (WB)	43 kDa
**Wilms Tumor Protein**	Abcam #ab52933	1:100 (IF)	----
		1:500 (WB)	51kDa
	R&D AF5729	2.5mg/ml (FC)	----
	Abcam #89901	1:100 (FC mouse)	----
**Zonal Occludens 1**	Abcam #ab59720	1mg/ml (IF)	----

<u>Supplementary Tables S2-S6</u>

Summary Tables for Ingenuity Pathway Analysis of Microarrays

A detailed list of genes compared for differential expression between undifferentiated AKPC-P and de-differentiated hIPod and between differentiated hAKPC-P and re-differentiated hIPod. All fold variations listed are statistically significant (P<0.05).

Table S2 – Summary table reporting fold variation for podocyte and slit diaphragm markers A detailed list of podocyte and slit diaphragm genes compared for differential expression between de-differentiated hIPod and undifferentiated hAKPC-P (on the left, light grey) and between re-differentiated hIPod and differentiated hAKPC-P (on the right, dark grey). Positive values indicate a higher gene expression in the hIPod versus the hAKPC-P, while negative values indicate a higher gene expression in hAKPC-P. All fold variations listed are statistically significant (P<0.05).

PODOCYTE AND SLIT DIAPHRAGM MARKERS							
hAKPC-P undifferentiated versus hIPod de-differentiated				hAKPC-P differentiated versus hIPod re-differentiated			
Gene	Fold Change	Gene	Fold Change	Gene	Fold Change	Gene	Fold Change
ACTN4	---	LRRC7	3.51	ACTN4	---	LRRC7	3.51
CD2AP	1.44	NPHS1	-1.26	CD2AP	1.44	NPHS1	-1.26
CD36	1.02	NPHS2	---	CD36	1.02	NPHS2	---
CDH3	-1.35	PDPN	-1.40	CDH3	-1.35	PDPN	-1.40
CHKA	2.29	PLCE1	-1.39	CHKA	2.29	PLCE1	-1.39
CR1	-1.13	PODXL	-4.78	CR1	-1.13	PODXL	-4.78
CTNNB1	1.13	PTPRO	-1.08	CTNNB1	1.13	PTPRO	-1.08
EFNB1	-2.44	SYNPO	-1.38	EFNB1	-2.44	SYNPO	-1.38
EZR	-1.02	TJP1	1.32	EZR	-1.02	TJP1	1.32
F Actin	---	TRPC6	---	F Actin	---	TRCP6	---
FAT1	-1.49	WT1	1.19	FAT1	-1.49	WT1	1.19
LMX1B	-1.11			LMX1B	-1.11		

Table S3 – Summary table reporting fold variation for markers for GBM formation A detailed list of GBM related genes compared for differential expression between de-differentiated hIPod and undifferentiated hAKPC-P (on the left, light grey) and between re-differentiated hIPod and differentiated hAKPC-P (on the right, dark grey). Positive values indicate a higher gene expression in the hIPod versus the hAKPC-P, while negative values indicate a higher gene expression in hAKPC-P. All fold variations listed are statistically significant (P<0.05).

GLOMERULAR BASEMENT MEMBRANE MARKERS							
hAKPC-P undifferentiated versus hIPod de-differentiated				hAKPC-P differentiated versus hIPod re-differentiated			
**Gene**	Fold Change	**Gene**	Fold Change	**Gene**	Fold Change	**Gene**	Fold Change
AGRN	-1.37	ITGAV	-1.41	AGRN	---	ITGAV	6.28
CDH1	-1.13	ITGB1	-1.29	CDH1	-2.37	ITGB1	3.17
COL4A1	-1.68	ITGB3	-1.58	COL4A1	2.36	ITGB3	20.80
COL4A2	-1.21	LAMA1	1.36	COL4A2	2.87	LAMA1	---
COL4A3	-1.07	LAMA5	-2.46	COL4A3	-2.80	LAMA5	-2.67
COL4A4	-1.14	LAMB1	-1.28	COL4A4	-2.56	LAMB1	6.84
COL4A5	-1.80	LAMB2	-2.28	COL4A5	-1.95	LAMB2	---
ELN	-1.18	LAMC1	-1.15	ELN	-2.77	LAMC1	7.18
FN1	-1.06	MPV17	-1.49	FN1	19.65	MPV17	6.27
HSPG2	---	MYO1E	-1.14	HSPG2	---	MYO1E	2.12
ILK	---	NID1	1.50	ILK	1.33	NID1	---
ITGA1	1.37	NID2	-7.26	ITGA1	9.40	NID2	---
ITGA3	-1.42	WT1	1.19	ITGA3	8.16	WT1	-1.62
ITGA5	-2.16			ITGA5	2.58		

Table S4 – Summary table reporting fold variation for markers involved in cell cycle regulation in podocytes A detailed list of cell cycle related genes compared for differential expression between de-differentiated hIPod and undifferentiated hAKPC-P (on the left, light grey) and between re-differentiated hIPod and differentiated hAKPC-P (on the right, dark grey). Positive values indicate a higher gene expression in the hIPod versus the hAKPC-P, while negative values indicate a higher gene expression in hAKPC-P. All fold variations listed are statistically significant (P<0.05).

CELL CYCLE MARKERS							
hAKPC-P undifferentiated versus hIPod de-differentiated				hAKPC-P differentiated versus hIPod re-differentiated			
**Gene**	Fold Change	**Gene**	Fold Change	**Gene**	Fold Change	**Gene**	Fold Change
ATM	----	Cyclin B	----	ATM	13.19	Cyclin B	----
ATR	----	Cyclin D	----	ATR	----	Cyclin D	----
CCNH	1.36	Cyclin E	----	CCNH	2.96	Cyclin E	----
CDC25A	2.21	DP1-E2F-Rb	----	CDC25A	----	DP1-E2F-Rb	----
CDK1	1.99	E2f	----	CDK1	----	E2f	----
CDK2	1.46	GSK3B	----	CDK2	4.01	GSK3B	2.14
CDK4/6	----	Hdac	----	CDK4/6	----	Hdac	----
CDK7	1.40	MYT1	-1.17	CDK7	2.70	MYT1	-3.00
CDKN1A	-2.90	PP2A	----	CDKN1A	6.89	PP2A	----
CDKN1B	1.35	RAF1	1.18	CDKN1B	2.83	RAF1	----
CDKN1C	----	RB1	1.14	CDKN1C	-2.68	RB1	29.82
CDKN2A	1.65	Scf	----	CDKN2A	6.84	Scf	----
CDKN2B	----	TFDP1	1.41	CDKN2B	----	TFDP1	11.27
CDKN2C	1.42	Tgf beta	----	CDKN2C	-3.02	Tgf beta	----
CDKN2D	----	TP53	1.21	CDKN2D	----	TP53	9.81
Cyclin A	----	WEE1	1.57	Cyclin A	----	WEE1	19.11

Table S5 – Summary table reporting fold variation for markers involved in Ca^{++} signaling in podocytes A detailed list of Ca^{++} signaling related genes compared for differential expression between de-differentiated hIPod and undifferentiated hAKPC-P (on the left, light grey) and between re-differentiated hIPod and differentiated hAKPC-P (on the right, dark grey). Positive values indicate a higher gene expression in the hIPod versus the hAKPC-P, while negative values indicate a higher gene expression in hAKPC-P. All fold variations listed are statistically significant (P<0.05).

Ca++/K+/Na+ SIGNALING MARKERS							
hAKPC-P undifferentiated versus hIPod de-differentiated				hAKPC-P differentiated versus hIPod re-differentiated			
**Gene**	Fold Change	**Gene**	Fold Change	**Gene**	Fold Change	**Gene**	Fold Change
AKAP5	1.74	KCNMA1	-3.89	AKAP5	----	KCNMA1	3.05
Alpha actin	----	KCNQ1	----	Alpha actin	----	KCNQ1	-3.68
ASPH	----	LETM1	-1.07	ASPH	----	LETM1	----
ATP1A2	-1.11	MAGI1	----	ATP1A2	-3.55	MAGI1	-1.85
ATP2A1	----	MCU	----	ATP2A1	----	MCU	4.48
ATP2A2	----	MEF2	----	ATP2A2	6.78	MEF2	----
ATP2C1	-1.15	MICU1	----	ATP2C1	6.07	MICU1	5.22
CABIN1	-1.20	MICU1-MCU	----	CABIN1	-1.83	MICU1-MCU	----
CACNA1C	----	Myosin	----	CACNA1C	-4.54	Myosin	----
CACNA1H	-1.16	Ncx	----	CACNA1H	-3.09	Ncx	----
CACNG1	-1.11	nAChRs	----	CACNG1	-3.12	nAChRs	----
Calcineurin protein(s)	----	NMDA Receptor	----	Calcineurin protein(s)	----	NMDA Receptor	----
Calmodulin	----	NPHS1	-1.26	Calmodulin	----	NPHS1	-3.66
CALR	-1.13	Pka	----	CALR	3.47	Pka	----
CaMK I	----	Pmca	----	CaMK I	----	Pmca	----
CAMK2D	-1.79	Rap	----	CAMK2D	2.15	Rap	----
CAMK4	4.00	Ryr	----	CAMK4	2.46	Ryr	----
CaMKII	----	RYR1	-1.11	CaMKII	----	RYR1	-3.34
Camkk	----	RYR2	-1.08	Camkk	----	RYR2	-2.67
CASQ	----	RYR3	-1.15	CASQ	----	RYR3	-2.74
CHP	-1.21	SCN4A	-1.21	CHP	6.07	SCN4A	-3.28
CLCN1	-1.06	SERCA	----	CLCN1	-2.62	SERCA	----
Creb	----	SLC24A6	1.09	Creb	----	SLC24A6	----
ERK1/2	----	SLC5A3	-2.66	ERK1/2	----	SLC5A3	-1.30
FXYD1	-1.09	Tni	----	FXYD1	-3.78	Tni	----
GRIA	----	TRDN	-1.02	GRIA	----	TRDN	-1.70
GRIK1	----	Tropomyosin	----	GRIK1	-2.69	Tropomyosin	----
Hdac	----	Troponin C	----	Hdac	----	Troponin C	----
HTR3A	-1.05	Troponin t	----	HTR3A	-3.27	Troponin t	----
ITPR	----	TRP	----	ITPR	----	TRP	----
KCNB2	1.27	TRPC3	1.29	KCNB2	-2.11	TRPC3	----
KCNE2	-1.33	TRPC6	----	KCNE2	-3.21	TRPC6	-2.58
KCNJ12	-1.12	VG Ca2++ channels	----	KCNJ12	-2.70	VG Ca2++ channels	----

Table S6 – Summary table reporting fold variation for contractility markers in podocytes A detailed list of contractility genes compared for differential expression between de-differentiated hIPod and undifferentiated hAKPC-P (on the left, light grey) and between re-differentiated hIPod and differentiated hAKPC-P (on the right, dark grey). Positive values indicate a higher gene expression in the hIPod versus the hAKPC-P, while negative values indicate a higher gene expression in hAKPC-P. All fold variations listed are statistically significant ($P<0.05$).

CONTRACTILITY MARKERS							
hAKPC-P undifferentiated versus hIPod de-differentiated				hAKPC-P differentiated versus hIPod re-differentiated			
Gene	Fold Change	Gene	Fold Change	Gene	Fold Change	Gene	Fold Change
ACTA1	----	MYH9	----	ACTA1	-3.30	MYH9	3.33
ACTA2	-1.63	MYL1	----	ACTA2	-1.42	MYL1	-2.45
ACTC1	1.10	MYL2	-1.18	ACTC1	-2.86	MYL2	----
ACTG2	----	MYL3	----	ACTG2	-4.28	MYL3	-3.44
ACTN2	----	MYL4	-1.11	ACTN2	-2.69	MYL4	-3.10
ACTN3	----	MYL5	-1.20	ACTN3	----	MYL5	-2.76
ACTN4	----	MYL6	-1.18	ACTN4	1.62	MYL6	-1.79
AGT	-1.08	MYL6B	----	AGT	----	MYL6B	----
ANXA6	-1.20	MYL9	----	ANXA6	3.13	MYL9	1.68
ARHGAP24	-1.83	MYLK	----	ARHGAP24	-1.45	MYLK	----
ARID5B	-1.71	MYLK2	----	ARID5B	----	MYLK2	-3.30
Arp2/3	----	MYLPF	1.01	Arp2/3	----	MYLPF	-2.62
AVP	----	MYO10	-1.53	AVP	----	MYO10	2.61
C3	1.53	MYO1B	-1.15	C3	12.61	MYO1B	9.19
CALCB	-1.52	MYO1C	-1.10	CALCB	-3.08	MYO1C	3.29
CAPZA1	1.07	MYO1D	3.02	CAPZA1	1.72	MYO1D	----
CAPZA2	----	MYO1E	-1.14	CAPZA2	-1.39	MYO1E	2.12
CAPZB	-1.09	MYO6	1.23	CAPZB	2.42	MYO6	7.23
CASQ1	----	MYOCD	1.01	CASQ1	-3.12	MYOCD	4.38
CAV1	-1.52	MYOF	-1.04	CAV1	33.76	MYOF	17.44
CAV3	-1.44	MYOM1	-1.04	CAV3	-3.09	MYOM1	-2.57
CD2AP	1.44	MYOM2	-1.04	CD2AP	10.04	MYOM2	-2.79
CNN1	1.95	Myosin2	----	CNN1	----	Myosin2	----
COL4A3BP	-1.07	MYOT	----	COL4A3BP	1.77	MYOT	-3.05
CTGF	-1.14	NEB	----	CTGF	4.63	NEB	----
CTSL2	1.36	NKX2-5	-1.11	CTSL2	----	NKX2-5	-2.30
CTTN	-1.18	NMU	-1.23	CTTN	4.01	NMU	-2.33
CXCR4	-1.02	NOS1	-1.05	CXCR4	-2.00	NOS1	-2.72
DES	----	NPHS1	-1.26	DES	-3.06	NPHS1	-3.66
DTNA	----	NPHS2	----	DTNA	----	NPHS2	-2.81
EDN1	-2.00	PAR3-PAR6-aPKC	----	EDN1	9.06	PAR3-PAR6-aPKC	----
EDN2	-1.57	PLCE1	-1.39	EDN2	-2.78	PLCE1	4.61
EGF	1.58	PXN	-1.60	EGF	-1.90	PXN	----
ELANE	-1.00	RHOA	1.03	ELANE	-3.39	RHOA	1.63
EZR	-1.02	ROCK1	1.73	EZR	4.15	ROCK1	10.32
F Actin	----	ROCK2	1.10	F Actin	----	ROCK2	3.01
FKBP1A	-1.31	RRAD	1.10	FKBP1A	5.14	RRAD	1.54
FLT1	-1.21	SGCA	-1.11	FLT1	-2.22	SGCA	-3.30
GJA1	-2.54	SLN	1.62	GJA1	10.16	SLN	----
GJC1	----	SMAD5	1.05	GJC1	----	SMAD5	7.26
HOMER1	----	SMPX	----	HOMER1	----	SMPX	----
HSBP1	1.62	SMTN	-1.49	HSBP1	4.04	SMTN	1.52
ID1	-1.68	SNTA1	1.05	ID1	----	SNTA1	-2.14
ID2	-1.72	SNTB1	1.10	ID2	----	SNTB1	2.06
ID3	-1.36	SPHK1	1.40	ID3	----	SPHK1	1.65
IGF1	-1.26	SRF	----	IGF1	-3.87	SRF	-2.23
ILK	----	SRI	-1.08	ILK	1.33	SRI	15.44
INF2	----	SRSF1	1.13	INF2	----	SRSF1	-1.64
ITGA2	-1.22	SYNPO	-1.38	ITGA2	12.32	SYNPO	-1.99
JSRP1	-1.12	TAC3	-1.11	JSRP1	-3.18	TAC3	-2.77
KNG1	----	Talin	----	KNG1	----	Talin	----
MAP2K1	-1.32	TNNC1	-1.10	MAP2K1	3.34	TNNC1	-3.74
MAP2K3	----	TNNC2	-1.15	MAP2K3	-1.34	TNNC2	-3.64
MAP2K6	-1.29	TNNI1	-1.14	MAP2K6	-3.94	TNNI1	-3.40
MBNL1	1.27	TNNI2	-1.10	MBNL1	-1.37	TNNI2	-3.68
MMP1	-10.88	TNNI3	-1.03	MMP1	-1.33	TNNI3	-2.67
MYBPC1		TNNT1	-1.37	MYBPC1	----	TNNT1	-1.71
MYBPC2	-1.16	TNNT2	-1.26	MYBPC2	-2.88	TNNT2	-4.69
MYBPC3	-1.02	TNNT3	-1.08	MYBPC3	-3.06	TNNT3	-3.61
MYBPH	-1.09	TPM1	----	MYBPH	-3.78	TPM1	----
MYH1	-1.06	TPM2	-1.73	MYH1	----	TPM2	1.55
MYH10	----	Tpm4	----	MYH10	1.72	Tpm4	----
MYH11	----	TRPV1	-1.13	MYH11	----	TRPV1	----
MYH13	-1.12	TTN	-1.09	MYH13	-3.04	TTN	-2.84
MYH14	-1.06	UCN	-1.19	MYH14	-3.71	UCN	-3.23
MYH2	-1.26	UTRN	-1.02	MYH2	-2.43	UTRN	5.28
MYH4	-1.21	UTS2	----	MYH4	-2.87	UTS2	----
MYH6	-1.08	VCL	-1.10	MYH6	-3.14	VCL	3.39
MYH7	----	VEGFB	----	MYH7	-2.37	VEGFB	----
